# A Bacterial Ras-Like Small GTP-Binding Protein and Its Cognate GAP Establish a Dynamic Spatial Polarity Axis to Control Directed Motility

**DOI:** 10.1371/journal.pbio.1000430

**Published:** 2010-07-20

**Authors:** Yong Zhang, Michel Franco, Adrien Ducret, Tâm Mignot

**Affiliations:** 1 Institut de Microbiologie de la Méditerranée–Université Aix-Marseille-Laboratoire de Chimie Bactérienne, Marseille, France; 2 State Key Laboratory of Microbial Technology, College of Life Science, Shandong University, Jinan, China; 3 Institut de Pharmacologie Moléculaire et Cellulaire–Université de Nice-Sophia Antipolis, Valbonne, France; Massachusetts Institute of Technology, United States of America

## Abstract

Regulated cell polarity is central to many cellular processes. We investigated the mechanisms that govern the rapid switching of cell polarity (reversals) during motility of the bacterium *Myxococcus xanthus*. Cellular reversals are mediated by pole-to-pole oscillations of motility proteins and the frequency of the oscillations is under the control of the Frz chemosensory system. However, the molecular mechanism that creates dynamic polarity remained to be characterized. In this work, we establish that polarization is regulated by the GTP cycle of a Ras-like GTPase, MglA. We initially sought an MglA regulator and purified a protein, MglB, which was found to activate GTP hydrolysis by MglA. Using live fluorescence microscopy, we show that MglA and MglB localize at opposite poles and oscillate oppositely when cells reverse. In absence of MglB, MglA-YFP accumulates at the lagging cell end, leading to a strikingly aberrant reversal cycle. Spatial control of MglA is achieved through the GAP activity of MglB because an MglA mutant that cannot hydrolyze GTP accumulates at the lagging cell end, despite the presence of MglB. Genetic and cell biological studies show that the MglA-GTP cycle controls dynamic polarity and the reversal switch. The study supports a model wherein a chemosensory signal transduction system (Frz) activates reversals by relieving a spatial inhibition at the back pole of the cells: reversals are allowed by Frz-activated switching of MglB to the opposite pole, allowing MglA-GTP to accumulate at the back of the cells and create the polarity switch. In summary, our results provide insight into how bacteria regulate their polarity dynamically, revealing unsuspected conserved regulations with eukaryots.

## Abstract 2

Directional control of bacterial motility is regulated by dynamic polarity inversions driven by pole-to-pole oscillation of a Ras family small G-protein and its associated GTPase-activating protein.

## Author Summary

Motile cells have evolved complex regulatory networks to respond to environmental cues and change their direction of movement appropriately. In this process, an arsenal of receptor-coupled small G-proteins acts as a cellular compass to dynamically polarize the leading edge and regulate the motility response. However, the precise mechanism of action of these G-proteins in controlling bacteria movement on solid surfaces has remained an enigma. We investigate this process in Gram negative *Myxococcus xanthus* cells. Surprisingly, we find that the Ras-like small G-protein MglA polarizes the cell by accumulating at the leading cell pole in its active GTP-bound form. This localization is dependent on MglB, a GTPase-activating protein that converts MglA to its inactive form specifically at the opposite, lagging cell pole. Furthermore, we show that a receptor-coupled signal transduction cascade can activate re-localization of MglA and MglB at opposite poles in a synchronous manner, resulting in inversion of the polarity axis and cell movement in the opposite direction. Thus, a simple, eukaryote-like signaling module also governs dynamic polarity mechanisms in bacteria, demonstrating broader conservation of these signaling systems than initially suspected.

## Introduction

In living cells, environmental changes and cell-cell regulations require transient cellular processes relying on the ability of the cells to regulate their underlying ultrastructures dynamically. For example, during chemotaxis, eukaryotic cells sense and migrate towards a chemical gradient, which requires complex spatial regulation of actin cytoskeleton dynamics [1]. The cells use a directional sensing system as a compass to favor the formation of pseudopodia towards or away from a source of chemoattractrant or repellent [1]. In this process, the cell adopts a polarized morphology, to define a front and a rear to coordinate actin polymerization at the leading edge with contractile forces generated by myosin motors at the rear. The actin cytoskeleton and the membrane are rearranged by a complex signaling network involving Receptor/G-proteins and, centrally, small GTPases of the Ras superfamily [2]. For example, in leukocytes, or in the amoeba *Dictyostelium discoidum*, polarization is achieved by a complex interplay of multiple small GTP-binding proteins at the front and the rear involving Ras, Rac, Cdc42, and Rho (see [2] for a detailed review of these regulations).

Owing to the small size of the bacterial cell, it is generally accepted that dynamic processes such as motility are regulated at the temporal rather than at the spatial level. For example, chemotaxis in liquid media relies on a temporal signal transduction cascade that switches the rotation of the flagellum [3]. However, one conspicuous example of dynamic cell polarization occurs during *Myxococcus xanthus* motility over solid surfaces (gliding motility): rod-shaped *myxococcus* cells control their direction of movement by inverting their polarity, rapidly switching their leading pole into their lagging cell pole (cellular reversal) [4]. Cellular reversals are highly regulated and mutants with impaired reversal frequencies cannot accomplish complex multicellular behaviors such as predation [5] and the capacity to develop fruiting bodies [6].

Cellular reversals imply that the directionality of the motility machinery can be rapidly inverted. In *Myxococcus*, two motility engines power locomotion: the first motility engine, a type-IV pilus, polymerizes fibers at the leading cell pole, which act as “grappling hooks” as they extend and retract to pull the cell forward [7]. The pili constitute the so-called Social (S)-motility system because they are involved in the movement within large cell groups, presumably because they allow tight cell-cell interactions [8]; the second motility system is not as well characterized and involves dynamic eukaryotic-like focal adhesions and the secretion of a polysaccharidic slime [9]. This system is usually referred to as the Adventurous (A)-motility system because in contrast to the pili, it promotes the movement of single isolated cells [10]. Recently, (A)- and (S)-motility components have been tracked in live gliding cells by time lapse fluorescence microcopy using chimeric fluorescent reporter proteins. Core type-IV pilus sub-units were found to be pre-assembled at both cell poles, but some key subunits, the extension and retraction ATPases and the FrzS protein, shuttled from pole-to-pole and were only clustered at one pole, suggesting that reversals occur following completion of an active pilus machine at the leading cell pole [11],[12]. Likewise, the A-motility focal adhesions, visualized by the AglZ-YFP protein, are assembled at the leading cell pole and are slowly moved to the rear where they are dispersed; at the time of reversal, the existing AglZ clusters are first dispersed and then reassembled from the new leading pole [13]. A-motility also involves proteins that accumulate and switch at the back of the cell (RomR), showing that motility requires both a head and a tail [14]. The two motility systems must be coordinated not to counteract each other, meaning that their directionality must be switched together when the cell reverses. Consistent with this, FrzS (S-motility) and RomR (A-motility) have been shown to oscillate synchronously [14].

Synchronous pole-pole oscillations of proteins belonging to the (A)- and (S)-motility are regulated by the Frz chemotaxis-like system [12],[13],[14]. The Frz core components involve a cytosolic chemoreceptor-like protein FrzCD, its coupling protein FrzA, and a cognate histidine kinase FrzE [6]. Auto-phosphorylation of FrzE following receptor activation allows transfer of phosphoryl groups to the downstream response regulator FrzZ [15],[16]. *frz*-null mutations decrease the cellular reversal frequency dramatically, whereas *frz*-gain of function mutations (*frzCD^c^*) increase the reversal frequency. Consistent with this, both classes of mutations respectively abolish or increase spatial oscillations of the (A)- and (S)-motility protein reporters [12],[13]. It was suggested that the Frz system constitutes a biochemical oscillator to regulate a downstream spatial oscillator, thus acting as a molecular clock to finely tune the reversal frequency to the ever-changing environmental conditions [17]. However, proving this attractive hypothesis will require extended characterization of the regulation mechanism.

How is the cell dynamically polarized to target motility proteins to opposite cell poles when cells reverse? A likely candidate for such regulation is the MglA protein, a bacterial small G-protein of the Ras-superfamily [18]. Previously, it was shown that MglA interacts directly with FrzS and AglZ and that FrzS, AglZ, and RomR are mislocalized in an *mglA* mutant [14],[18],[19]. Assembly of the focal adhesion clusters specifically requires MglA to cooperate with the MreB actin cytoskeleton [18]. A difficulty is that MglA is required for the functionality of the motility engines themselves; thus, it could not be determined if MglA also has a role in directional control.

In this work, we investigated the role of the MglA GTP cycle and found that MglA acts as a cornerstone to coordinate spatial assembly and activity of the motility engines. We found that the establishment of a dynamic polarity axis relies directly on the sequestration of the MglA GTP-bound form at the leading cell pole and characterized a novel GTPase Activating Protein responsible for this spatial regulation.

## Results

### MglB Is a Guanine Nucleotide Hydrolysis Activating Protein (GAP) for MglA

**Figure 1:**
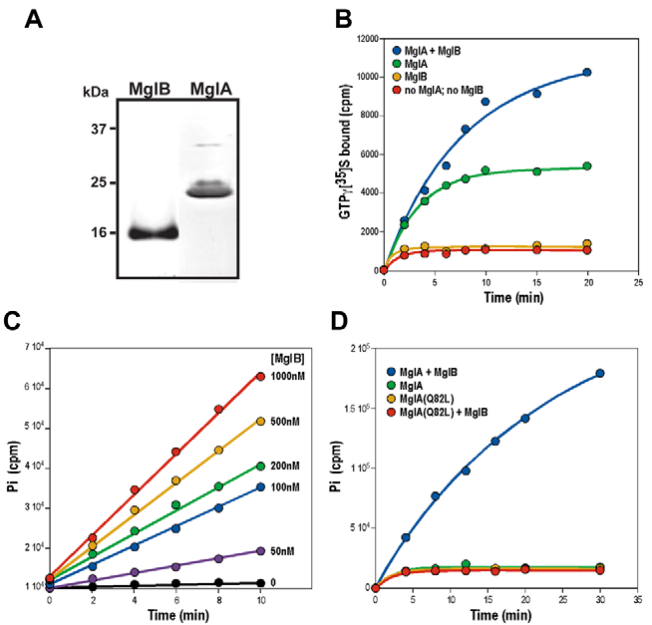
MglB is a guanine nucleotide hydrolysis activating protein for MglA. (A) Coomassie-stained gel showing purified MglA and MglB used in the biochemical assays. (B) MglB does not bind to non-hydrolysable GTPγS but stabilizes binding of MglA to GTPγS. Purified MglA (1 µM) and MglB (1 µM) were tested for their ability to bind GTPγS as described in experimental procedures. The presence of MglB does not accelerate the binding of GTPγS to MglA but increases the total amount of MglA bound to GTPγS by a factor of ∼2-fold. (C) Dose dependent activation of the MglA GTPase activity by MglB. Hydrolysis of GTP was measured by measuring ^32^Pi release as described in experimental procedures. (D) The Q82L mutation renders MglA insensitive to the GAP activity of MglB. GTP hydrolysis by MglA and MglA_Q82L_ following MglB addition was measured over time as in (C).

In eukaryotic cells, small G-proteins are critically regulated by Guanine nucleotide Exchange Factors (GEFs) and GTPase Activating Proteins (GAPs) [20],[21]. We have previously established that MglA can hydrolyze GTP, albeit at very slow rates in vitro, suggesting that an MglA-regulator with GAP activity could exist [18]. Based on sequence conservation, extensive search of the *Myxococcus* genome did not reveal eukaryotic-like regulators. Yet MglA is co-expressed with MglB, the founder member of a family of proteins that contains a so-called roadblock domain [22],[23]. The function of roadblock domains has not been demonstrated experimentally, but bioinformatics suggested that they might regulate the activity of a cognate NTPase [23]. Thus, MglB and MglB-like proteins could be prokaryotic regulators of bacterial small G-proteins such as MglA. To test this possibility, we purified recombinant MglA and MglB proteins to analyze in vitro whether MglB could act as a GAP for MglA (Figure 1A). In vitro, MglA bound but did not significantly hydrolyze radio-labeled GTP (Figure 1B and 1C). This result is consistent with previous results, showing that MglA hydrolyzed GTP at an extremely low rate in an enzyme-coupled assay [18]. In marked contrast, addition of MglB stimulated GTP hydrolysis by MglA in a dose-dependent manner (Figure 1C). MglB alone did not bind radioactive GTP (Figure 1B). Additionally, MglB was not found to affect GDP/GTP exchange on MglA: MglB stabilized the GTP bound form slightly (like a classical G-protein effector, Figure 1B) but did not modify the GDP off rate of MglA (unpublished data). Thus, MglB is an MglA GAP, which functions by switching MglA-GTP to MglA-GDP.

### MglB Is an Inhibitor of Cellular Reversals

**Figure 2:**
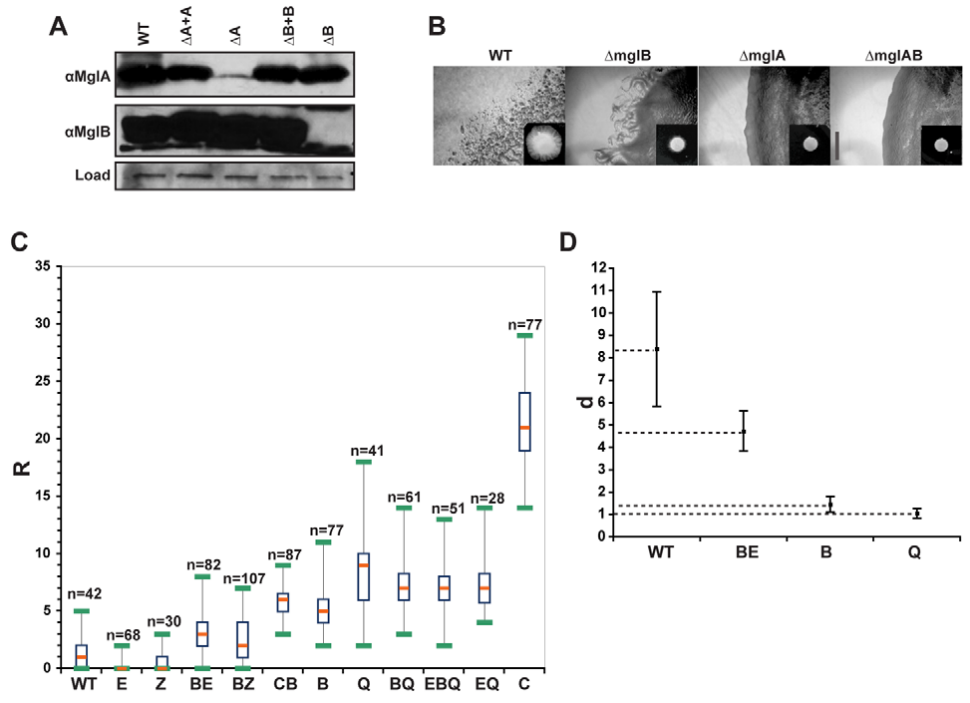
MglA is the most downstream component of a pathway involving Frz and MglB. (A) Levels of MglA and MglB expression in WT, *ΔmglA* (ΔA), *ΔmglB* (ΔB), and the respective complemented (ΔA+A, ΔB+B) strains assayed by Western blotting with anti-MglA and anti-MglB antibodies. (B) Motility phenotypes of WT, *ΔmglB*, *ΔmglA*, and *ΔmglAB* strains. Colony edges after 48 h incubation on hard (1.5%) agar are shown. Insets: colonies on soft (0.5%) agar, a substratum that only allows S-motility, visible at the edges. Scale bar = 1 mm. (C) Box plots of measured reversal frequencies in the various strains. The orange bar represents the average reversal frequency of each population. R: number of reversals scored in 30 min. E: *ΔfrzE*, Z: *ΔfrzZ*, BE: *ΔmglB ΔfrzE*, BZ: *ΔmglB ΔfrzZ*, CB: *frzCD^c^ ΔmglB*, B: *ΔmglB*, Q: *mglA_Q82L_*, BQ: *ΔmglB mglA_Q82L_*, EBQ: *ΔfrzE ΔmglB mglA_Q82L_*, EQ: *ΔfrzE mglA_Q82L_*, C: *frzCD^c^*. n, number of cells that were tracked. (D) Traveled distances between reversals in different strains. d, ratio of the traveled distance over the cell length. A ratio of 1 indicates that the cells reverse after moving a distance corresponding to one cell length. Strains are labeled as in (C). *n* = 10 for each strain.

What is the function of MglB in vivo? mglAB are encoded within a putative operon. In a previous work, deletion of *mglB* resulted in a dramatic reduction of the MglA levels, which precluded in-depth study of the function of MglB [24]. MglB was proposed to have a chaperone function for MglA explaining the observed lack of MglA stability [24], yet the low levels of MglA expression could also have been due to polar effects of the *mglB* deletion. To test the function of *mglB* in vivo, we deleted the region encoding residues 10–159 of *mglB* (MglB contains 159 residues). To show that this *mglB* deletion created no downstream polar effects on the expression of *mglA*, we successfully complemented the Δ*mglB* mutant by integrating another copy of *mglB* at an ectopic site on the *Myxococcus* chromosome (the Mx8-phage attachment site, Figure 2A and Figure S1A). Likewise, deletion of *mglA* was fully complemented when *mglA* was expressed from the Mx8-phage attachment site (Figure S1B). Western blots using anti-MglA and anti-MglB antibodies showed that MglA and MglB were produced stably in the *ΔmglB* and *ΔmglA* mutant, respectively (Figure 2A). We conclude that MglA and MglB are stable independently from each other and that their respective functions can be studied with the deletion mutants.

We tested the motility of the *ΔmglB*, *ΔmglA*, and *ΔmglAB* mutants in the hard (testing both A- and S-motility) and soft (testing S-motility only) agar assays. Swarming of the *ΔmglB* mutant was severely defective but detectable on both substrata (Figure 2B). The *ΔmglA* and *ΔmglAB* mutants looked completely non-motile under all conditions, showing that *mglA* acts downstream from *mglB* (Figure 2B). The motility defect of the *ΔmglB* mutant may be due to defects of the motility engines, aberrant directional control, or both. Time-lapse analysis of *ΔmglB* motile cells revealed that the cells moved with WT velocities (unpublished data). Strikingly, the *ΔmglB* mutant displayed an altered reversal frequency and reversed their direction of movement more frequently than WT cells (Figure 2C). Thus, MglB acts upstream from MglA and inhibits cellular reversals.

### MglB Acts Downstream from the Frz-Signal Transduction Pathway

The Frz pathway regulates the reversal frequency of *Myxococcus* cells [25]. We wondered where MglB acts in the pathway. We combined *frzE*-null (kinase null) and *frzZ*-null (response-regulator null) mutations with a *ΔmglB* deletion and scored the reversal frequency of the double mutants. Strikingly, the *ΔmglB* mutation restored reversals of both the *ΔfrzZ* and *ΔfrzE* mutants (Figure 2C). The reversal frequency of the double mutants was significantly higher than the reversal frequency of WT cells yet remained slightly lower than the reversal frequency of the *ΔmglB* mutant (Figure 2C). To confirm the epistastic relationship between *mglB* and *frz*, we also combined a *frzCD^c^* mutation (a mutation that hyperactivates Frz signaling) with the *ΔmglB* deletion. Both mutants hyper-reverse but they have significantly distinct reversal frequencies: the *ΔmglB* mutant has an average frequency of ∼10 rev.hour^−1^, while the *frzCD^c^* has an average frequency of ∼40 rev.hour^−1^ (Figure 2C). A *frzCD^c^ ΔmglB* mutant reversed with frequencies that were indistinguishable from the *ΔmglB* mutant (∼10 rev.hour^−1^), confirming that *mglB* acts downstream from the Frz-pathway. Agar swarming assays showed that the swarming pattern of the *ΔfrzE ΔmglB* was almost identical to the swarming pattern of the *ΔmglB* mutant, confirming that *mglB* acts downstream from the Frz pathway (Figure S2).

To clarify whether the *ΔmglB* mutant is indeed distinct from the *ΔfrzE ΔmglB* mutant, we measured the average distances traveled by the cells between reversals (Figure 2D). We found that the *ΔfrzE ΔmglB* mutant cells moved on average a distance corresponding to 4–5 cell lengths before they reversed (versus ∼8 cell lengths for the WT, Figure 2D). On the contrary, the *ΔmglB* mutant cells almost systematically reversed after moving a distance corresponding to one cell length (Figure 2D, see below).

Taken together, these results suggest that the Frz pathway activates cellular reversals by relieving an inhibition that *mglB* exerts on *mglA*: low reversal frequencies in *frz*-null mutants can thus be explained by a failure to relieve MglB inhibition, a mechanism that depends on FrzZ. However, Frz must also be able to signal to MglA independently from MglB because Frz-dependent regulation (albeit highly abnormal) is still detected when the reversal frequency of *ΔmglB* mutant is compared to the reversal frequency of double *ΔfrzE ΔmglB* mutant (see Discussion). This branching in the signaling pathway must occur downstream from FrzZ because *ΔfrzZ ΔmglB* mutants reverse with frequencies that are similar to the *ΔfrzE ΔmglB* (Figure 2C).

### MglB Localizes at the Lagging Cell Pole and Oscillates in a Frz-Dependent Manner

**Figure 3:**
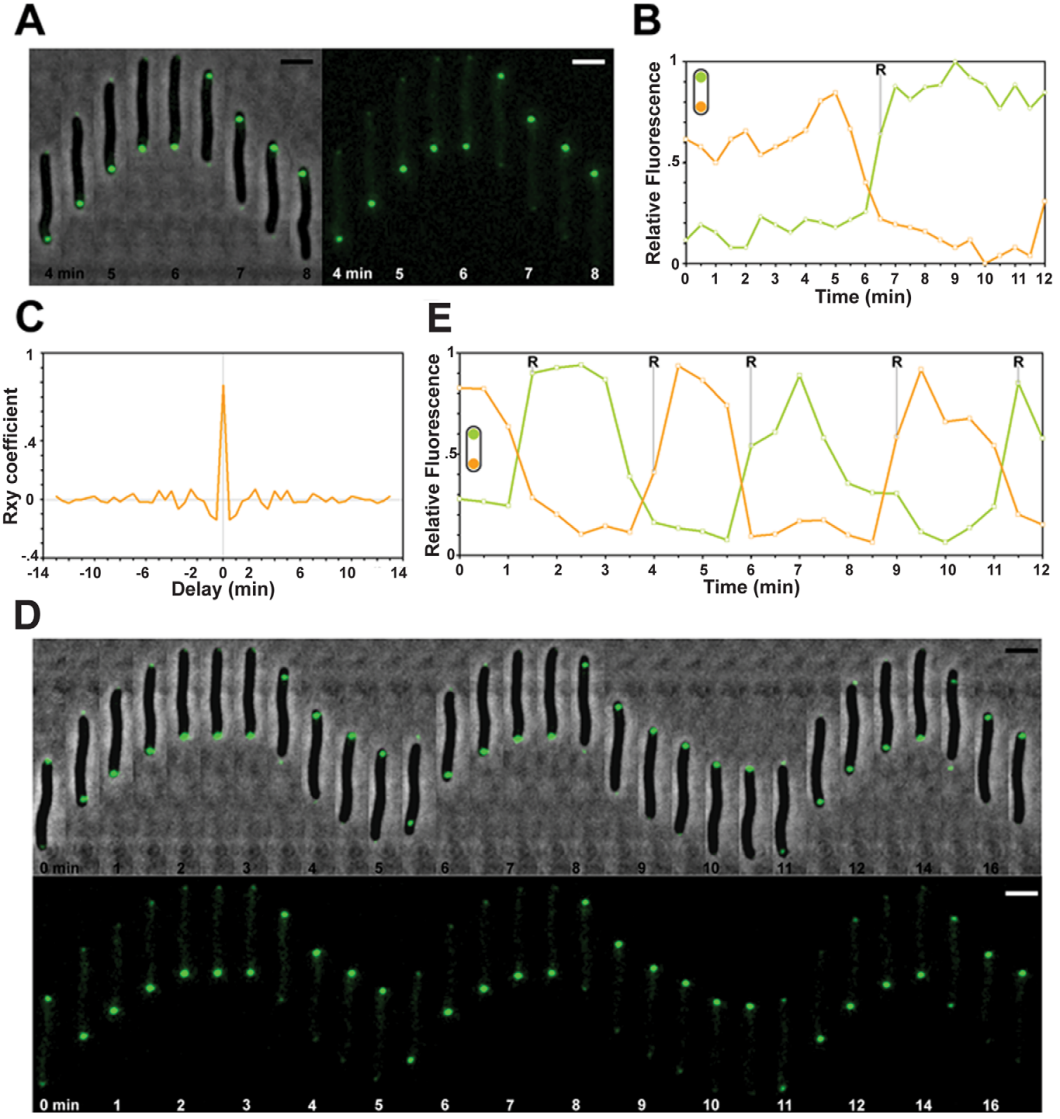
MglB-YFP localizes at the lagging cell pole. (A) MglB-YFP switches upon cellular reversals. Scale bar = 2 µm. (B) Quantification of the relative fluorescence at the poles for the cell shown in (A) over time (min). Green line: initial leading pole. Orange line: initial lagging pole. R: reversal. (C) cross-correlation between MglB-YFP oscillations and cell reversals. (D) MglB-YFP dynamics are regulated by Frz. Oscillations of MglB-YFP in a *frzCD^c^* mutant. Scale bar = 2 µm. (E) Quantification of the relative fluorescence at the poles for the cell shown in (D) over time (min). Green line: initial leading pole. Orange line: initial lagging pole. R: reversal.

To further understand the role of MglB, we generated a functional MglB-YFP fusion to investigate MglB dynamics during the reversal cycle (Figure S1). A single MglB-YFP focus was observed at the lagging cell pole and this focus switched systematically to the new lagging pole when cells reversed (Figures 3A, 3B, and S4A). Automated cross-correlation analysis confirmed that MglB oscillations are indeed coupled to cellular reversals (Figure 3C). Introduction of the *frzCD^c^* allele led to hyper-oscillations of MglB-YFP (Figure 3D and 3E) whereas a *frzE*-null mutation abolished oscillations (unpublished data), confirming that MglB is indeed regulated by Frz.

### MglA and MglB Oscillate Synchronously and Inversely

Our genetic and biochemical evidence suggests that MglB inhibits reversals by activating GTP hydrolysis by MglA at the lagging cell pole. In a previous work, we constructed a partially functional MglA-YFP chimera: cells expressing MglA-YFP alone were motile but significantly impaired in their reversal frequency, precluding studies of MglA dynamics during the reversal cycle (Figure S3 [18]). To monitor the dynamics of MglA-YFP in reversing cell, we expressed MglA-YFP in the presence of MglA. In these merodiploid cells, expression of MglA-YFP was not associated with detectable motility defects (see Protocol S1 for details on the construction and Figure S3). Thus, we conclude that MglA-YFP dynamics during reversal can be studied using the merodiploid system, which will be systematically used for the rest of this study.

**Figure 4:**
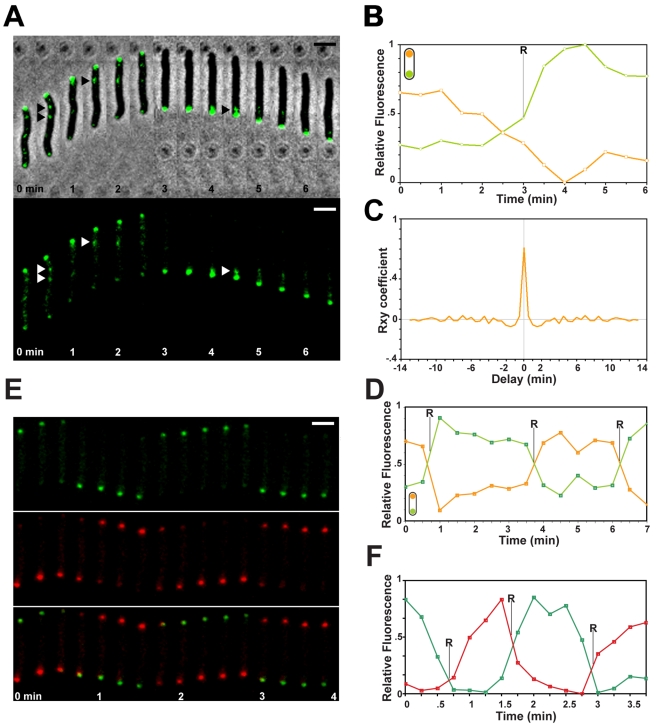
MglA and MglB oscillate inversely and synchronously. (A) MglA-YFP switches to the new leading pole when cells reverse. Fluorescence and corresponding phase contrast overlays are shown. Triangles show MglA internal clusters. Scale bar = 2 µm. (B) Quantification of the relative fluorescence (grey values, arbitrary units) at the poles for the cell shown in (A) over time (min). Orange line: initial leading pole. Green line: initial lagging pole. R: reversal. (C) Cross-correlation between MglA-YFP oscillations and cell reversals. (D) Dynamics of MglA-YFP in the *frzCD^c^* mutant. Legend reads as in (B). (E) Dynamics of MglA-YFP and MglB-mCherry in a reversing cell. Upper panel: MglA-YFP; middle panel: MglB-mCherry; lower panel: merge. Scale bar = 2 µm. (F) Quantification of the relative YFP (green) and mCherry fluorescence (red) at the initial leading pole for the cell shown in (E) over time (min).

In a fluorescent time-lapse motility assay, MglA-YFP localized at the leading cell and within fixed internal clusters (Figures 4A and S4B). MglA-YFP oscillated from pole to pole and was systematically redirected to the new leading pole at the time of reversal (Figure 4A and 4B). Cross-correlation analysis also confirmed that MglAYFP oscillations are coupled with the reversal cycle (Figure 4C). Thus, the localization of MglA-YFP is coupled to the reversal cycle. MglA-YFP dynamics were clearly regulated by the Frz pathway: in a *ΔfrzE* mutant oscillations of MglA-YFP were abolished (unpublished data), while they were enhanced and correlated with increased cellular reversals in a *frzCD^c^* mutant (Figures 4D and S5), confirming that MglA is a downstream component of the reversal switching machinery.

To monitor the dynamic behaviors of MglA and MglB simultaneously, we engineered merodiploid cells expressing both MglA-YFP and a functional MglB-mCherry. As expected from analysis of the individual fusions in WT and *frz* backgrounds, both proteins oscillated inversely and synchronously, switching to opposite poles when cells reversed (Figure 4F and 4E).

### MglB Is a Critical Determinant of Asymmetrical MglA-YFP Localization

**Figure 5:**
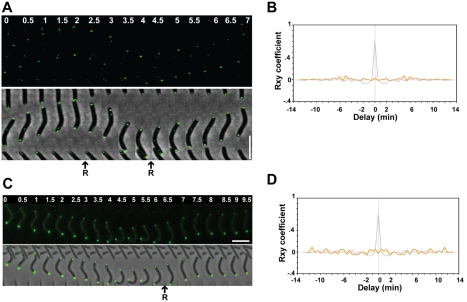
MglB prevents accumulation of MglA at the lagging cell pole. (A) MglA-YFP is bipolar in absence of *mglB*. Fluorescence and corresponding phase contrast overlays of a *ΔmglB* mutant expressing MglA-YFP are shown. Scale bar = 2 µm. (B) Cross-correlation between MglA-YFP dynamics and cell reversals in the Δ*mglB* mutant (orange curve) and the WT strain (gray curve). (C) MglA_Q82L_-YFP is bipolar. Fluorescence and corresponding phase contrast overlays of an *mglA_Q82L_* mutant expressing MglA_Q82L_-YFP are shown. Scale bar = 2 µm. (D) Cross-correlation between cell reversal and MglA_Q82L_-YFP dynamics (orange curve) or MglA-YFP (gray curve).

We then analyzed the dynamic localization of MglA-YFP in absence of MglB. In this mutant, MglA-YFP did not localize to one cell pole but localized to both cell poles: minor fluctuations in MglA-YFP fluorescence were observed over time, but these changes were rapid and transient and not obviously correlated with the timing of reversals (Figure 5A and 5B). Thus, a function of MglB is to prevent MglA-YFP accumulation at the lagging pole, which seems to result in aberrant reversals.

### MglB Regulates the Nucleotide State of MglA In Vivo

MglB could inhibit cellular reversals by catalyzing the transition from MglA-GTP to MglA-GDP at the lagging cell pole. The in vitro results show that MglA does not hydrolyze GTP significantly in absence of MglB; thus, MglA may be mostly GTP-bound in the *mglB* mutant. If so, a mutation that locks MglA in its GTP-bound form should mimic the *mglB* mutation. We designed an MglA mutant where Glutamine 82 is replaced by a Leucine, a mutation predicted to lock MglA in its GTP-bound state by inhibiting GTP hydrolysis (Figure S6A, [26]). In vitro, MglA_Q82L_ bound GTP stably like the WT MglA (Figure S6B), but contrarily to WT MglA and as expected, addition of MglB failed to activate hydrolysis, showing that MglA_Q82L_ cannot hydrolyze GTP (Figure 1D). When expressed in vivo, MglA_Q82L_ was found to be as stable as WT MglA (Figure S7).

If MglB prevents MglA binding at the lagging pole through its GAP activity, MglA_Q82L_ should localize at both cell poles despite the presence of MglB. As expected, an MglA_Q82L_-YFP chimera (see Protocol S1 for details on this construction) was mostly found at both cell poles and the cells reversed in the absence of MglA_Q82L_-YFP oscillations (Figure 5C and 5D), similarly to MglA-YFP in the *ΔmglB* mutant (Figure 5A and 5B). We conclude that the function of MglB is to catalyze the transition from MglA-GTP to MglA-GDP, preventing accumulation of MglA at the lagging cell pole and thus inhibiting reversal frequency.

### The MglA GTP Cycle Determines Reversal Frequency

MglA_Q82L_ expressing cells reversed with a reversal frequency that was almost identical to that of the *mglB* deletion mutant (Figure 2C and 2D). In fact, similarly to the *ΔmglB* mutant, *mglA_Q82L_* cells almost systematically moved exactly the length of one cell body before they reversed (Figure 2D). To confirm that reversals are regulated through the MglA GTP hydrolysis cycle, we measured the reversal frequencies of *mglA_Q82L_ ΔmglB* and *mglA_Q82L_ ΔmglB ΔfrzE* mutant cells. All mutants reversed with frequencies similar to the *mglA_Q82L_* (Figure 2C) showing that the MglA GTP switch is the final downstream output of the Frz MglB transduction pathway.

### The MglAB Module Creates Dynamic Cell Polarity

Our results suggest that the MglA and MglB proteins define the polarity switch that controls cellular reversals. However, an outstanding question remains: Why are cells still reversing in absence of a GTP hydrolysis cycle (for example in *ΔmglB* and *mglA_Q82L_* cells)? This seems paradoxical because a simple assumption was that in absence of dynamic polarization, the cells would be non-reversing rather than hyper-reversing. A clue, however, is that in all cases the cells reverse after they moved the distance of one cell length (Figure 2D).

**Figure 6:**
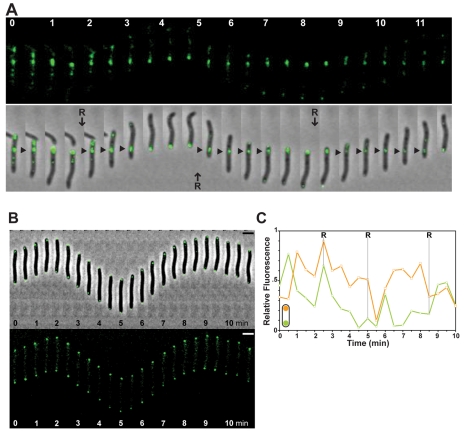
MglAB establish dynamic cell polarity. (A) AglZ-YFP dynamics in an *mglA_Q82L_* mutant. Triangles point to a major AglZ-YFP cluster that remains fixed at all times. Scale bar = 2 µm. (B) FrzS-YFP dynamics in an *mglA_Q82L_* mutant. Scale bar = 2 µm. (C) Quantification of the relative fluorescence at the poles for the cell shown in (B) over time (min). Orange line: initial leading pole. Green line: initial lagging pole.

Since single motile cells move essentially by A-motility, we analyzed AglZ-YFP dynamics in the *mglA_Q82L_* mutant. In WT cells, AglZ-YFP localizes at the leading pole and assembles within fixed adhesion clusters dispersed at the back of the cell (Figure S8 and [13]). In the *mglA_Q82L_* mutant, AglZ-YFP was not significantly retained at the cell pole; instead, a major fluorescent cluster remained at a fixed position relative to the substratum at all times (Figure 6A). Cells systematically reversed once the AglZ-YFP clusters accumulated at the lagging cell end (Figure 6A). Comparable results were obtained in the *mglB* mutant (unpublished data). Thus, AglZ-YFP no longer oscillates between poles in absence of the MglA GTP-cycle. Instead, we propose that the cells reverse because the A-motility system is intrinsically capable to switch its own directionality, for example, once key regulatory proteins such as AglZ accumulate at the lagging cell pole (see Discussion).

To confirm that the MglA GTP cycle is essential for dynamic cell polarity we also tracked the localization of the downstream S-motility protein FrzS-GFP in the *mglA_Q82L_* strain. Under our experimental conditions, single cells do not move by S-motility but S-motility proteins dynamics are still coupled to the directionality of A-motile cells [11],[12]. For example, FrzS-GFP clusters at the leading cell pole and oscillates from pole to pole during the reversal cycle in wild type cells ([12] and Figure S9A). In both the *mglA_Q82L_* and *ΔmglB* strains, FrzS-GFP localized to both cell poles but showed no detectable switching: fluorescence intensities fluctuated at the cell poles but these fluctuations did not correlate with the direction of movement (Figure 6B, 6C and Figure S9B). Finally, we also looked at the dynamics of the RomR-GFP protein, which belongs to the A-motility system and binds at the lagging end, oscillating inversely and synchronously with FrzS and AglZ [14]. In absence of MglB, RomR-GFP was bipolar and showed no oscillation, confirming the lack of dynamic cell polarity (Figure S10). All together, these results show that MglA and MglB establish a polarity axis that drives programmed cellular reversals.

## Discussion

GAP regulation has been reported to restrict active Ras-GTP spatially, for example, to control embryo polarization in *C. elegans*, budding site placement in yeast, and also directional control in *Dictyostelium* [27],[28],[29]. In all these cases, a specific GAP protein excludes the localization of Ras by catalytically activating the switch from GTP- to GDP-bound Ras. It is thus especially striking that the cellular regulatory mechanisms governing motility control in *M. xanthus*, a prokaryot, are conceptually similar. In this organism, a small G-protein (MglA) is spatially restricted to the leading cell pole in its GTP-bound form because its cognate GAP (MglB) excludes it from the opposite pole. We further show that this polarity axis can be rapidly inverted, providing a mechanism for directional motility control.

### How Does MglAB Create Dynamic Cell Polarity?

MglA-binding cues may in fact exist at both cell poles because MglA-GTP is bi-polar in absence of MglB regulation (i.e., MglA_Q82L_ or MglA in the *mglB* mutant). However, these cues are not dynamically regulated because bi-polar MglA does not oscillate with the reversal cycle. Also, FrzS and RomR localize non-dynamically to one cell pole in absence of MglA [14],[18]. Thus, targeting of motility proteins to the cell poles is probably wired into the cell cycle itself in a process similar to flagellar assembly at new division septa [30],[31]. Conceptually, polar curvature itself may play a role in recognition because small G-proteins and their regulators can bind curved membranes [32] and several proteins have been shown to recognize curvature at the bacterial poles [33],[34].

**Figure 7:**
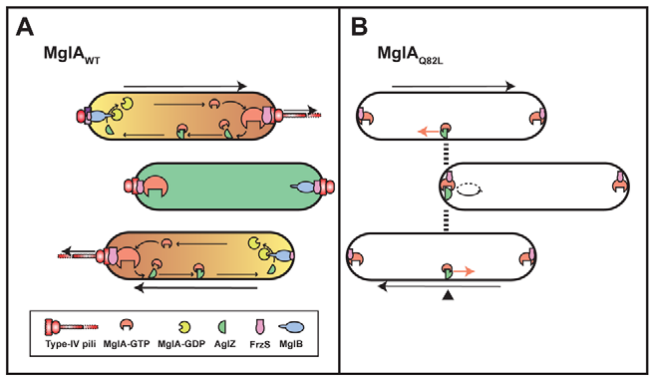
Working model for the regulation of dynamic cell polarity in *Myxococcus*. (A) Dynamic polarity switched during cellular reversals. See text for details. The orange yellow gradient symbolizes proposed MglA-GTP (orange) and MglA-GDP (yellow) distribution in the cell. At the time of reversal, an Frz-dependent unknown mechanism triggers MglA and MglB to switch to opposite poles. During the pause, the cell is depolarized, FrzS is symmetrically distributed at both cell poles, and the AglZ clusters are dispersed throughout the cytosol (green). The cycle is then reinitiated and the cell moves in the opposite direction. The sizes of the protein symbols reflect relative amounts at specific subcellular sites. (B) Cellular reversals in absence of an MglA GTP cycle. See text for details.

Several lines of evidence suggest that MglAB is the polarity generator that creates cellular reversals: (i) MglA acts downstream from the Frz pathway and switches systematically to the new leading pole. (ii) Expression of an MglA variant locked in its GTP-bound state is epistatic over *Δfrz* and *ΔmglB* mutations, showing that MglA is the most downstream component of the regulatory cascade that controls reversals. (iii) MglA interacts directly with FrzS and AglZ [18],[19] and is essential for the dynamic localization of FrzS, AglZ, and RomR. (iv) The perturbations of the MglA GTP cycle affect dynamic polarity of MglA itself and creates aberrant dynamic behaviors of the downstream proteins FrzS, AglZ, and RomR. (v) MglA is a *bona fide* small G-protein, a class of essential polarity regulators in eukaryotic cells. The results suggest that MglB acts to sequester MglA-GTP at the leading cell pole where it would activate both motility systems, for example, by engaging FrzS and AglZ (Figure 7A). Consistent with this, MglA_Q82L_-YFP, a GTP-locked mutant, accumulates at the lagging cell pole, despite the presence of MglB. MglB may also trigger dispersal of the focal adhesion clusters by inactivating cluster-associated MglA and preventing uncontrolled A-motility directional switches (see below). Thus, a Frz-dependent mechanism could simply invert the polarity axis by switching MglB to the opposite cell pole (Figure 7A).

This model is attractive but still has a number of unresolved questions. How are MglA and MglB switched to generate reversals? MglA- and MglB-YFP do not accumulate gradually at opposite poles but rather are rapidly relocated at the time of reversal (within ∼30 s) (Figures 3B and 4B), arguing that a transient signal input triggers the switch. Interestingly, bursts of phosphorylated FrzZ are predicted by mathematical modeling [17]. If true, FrzZ may trigger re-localization of MglB to the opposite cell pole directly, potentially by inhibiting the MglB GAP activity. However, this scenario is probably over-simplistic: our results clearly point to the existence of additional MglA regulators: the “pendulum” motility of the *ΔmglB* mutant can be suppressed partially by deletion of either *frzE* or *frzZ* (Figure 2D), suggesting the existence of an additional Frz-modulated regulator of MglA. *frz* suppression does not occur in cells expressing the MglA GTP-locked variant; thus, the suppression mechanism specifically occurs at the level of the MglA GTP cycle (Figure 2C). In addition, MglA may be mostly in its GTP-bound form in the *ΔmglB* mutant because the reversal frequency and localization of MglA-YFP were very similar in the *ΔmglB* and *mglA_Q82L_* mutants. Thus, *frz* suppression of the *ΔmglB* mutant pendulum motion argues that the MglA GTP-cycle is somewhat restored in the double mutant (albeit incompletely), for example because of an additional MglA GAP.

### MglB Defines a Novel Family of Bacterial GAPs

Eukaryotic small G-proteins are often controlled through the balancing actions of GAPs and GEFs, each catalyzing opposite steps of the GTP switch [21]. A GEF may be necessary to switch MglA back to the GTP bound form after MglB activation of GTP hydrolysis, but MglA could also spontaneously switch back to the GTP-bound state if the GTP/GDP balance is favorable in the cell. Small GTPase regulation by bacterial proteins is common during pathogenesis where invasive bacteria inject effectors that mimic GAPs and GEFs directly into the host cell to disrupt small GTPase signaling [35]. However, none of these virulence factors are related to MglB and there is no evidence that they have a physiological function in bacteria that express them. Thus, MglB-like proteins are probably major regulators of bacterial small GTPases. How MglB activates GTP hydrolysis by MglA is an interesting question because MglB contains a widespread so-called LC7/roadblock domain [23]. LC7/roadblocks are ancient protein domains conserved in all three eukaryotic, prokaryotic, and archeal kingdoms, yet very little information is available about their function. It was suggested that the members of this family regulate NTPases because proteins that contain roadblock domains are almost invariably linked to an NTPase [23]. For example, the LC7 protein is a conserved component of the Dynein light chain and has an important regulatory role on the activity of this motor [36]. However, the lack of a simple biochemical system to test LC7 function has hampered our understanding of how these domains work. Thus, understanding how MglB regulates MglA at the molecular level is potentially of great significance to understand the function of a widely conserved protein domain.

### The “Pendulum” Motion Highlights Unexpected Properties of the A-Motility Machinery

The oscillation dynamics of FrzS-GFP and RomR-GFP were abrogated in absence of the MglA-GTP cycle, suggesting that the MglAB proteins polarize the cell dynamically to regulate cellular reversals. Thus, we were surprised to find that the cells still reversed in absence of the MglA GTP-cycle. This was puzzling because the dynamics of RomR, an A-motility protein, are not coupled to the reversal cycle in the *ΔmglB* mutant. So how were these reversals generated if the cells are not dynamically polarized? Figure 7B depicts a proposed mechanism. In absence of the MglA GTP switch, active engine units may be assembled at the cell pole and produce movement. When these units reach the lagging cell end, they are not disassembled because MglB cannot activate MglA-GTP hydrolysis within the complexes. Instead, we propose that a threshold is reached at the pole, activating a built-in capacity in the machinery to reverse its directionality and resume movement in the opposite direction. The cycle can thus be repeated endlessly resulting in the “pendulum” behavior. The term “pendulum gliding” was borrowed from studies on Plasmodium sporozoites (apicomplexan parasites) [37]. We previously discussed that A-motility may be analogous to Apicomplexan gliding motility because the parasites assemble focal adhesion complexes at their apical end and glide forward by moving these adhesions rearward in an actomyosin motorized process [13],[38]. Strikingly, mutant sporozoites expressing a truncated version of the adhesion factor TRAP also move like a pendulum, most likely because they fail to relieve TRAP-dependent adhesions at their trailing end [37]. In *Myxococcus*, failure to relieve focal adhesions at the back of the cells could also trigger a switch to the opposite direction. Thus, a critical function of MglB is to suppress these periodic switches and allow the cell to move distances corresponding at least to several cell lengths before it reverses. The molecular basis for directional inversion needs investigation both in *Plasmodium* and *Myxococcus*.

In a previous work, we showed that A-motility complexes require the bacterial MreB actin cytoskeleton and MglA, much like focal adhesion complexes that drive eukaryotic cell migration [18]. Thus, the mechanisms underlying *Myxococcus* motility are remarkably similar to the mechanisms that drive eukaryotic cell motility. In *Dictyostelium discoidum*, chemotaxis involves a complex arsenal of receptor-activated small GTPases and their cognate regulators [1],[2]. In *Myxococcus*, polarity seems to be controlled by a single small G-protein switch that acts downstream of a chemotaxis-like signal transduction pathway. It is thus an ideal model system to dissect molecular regulations that may be extremely widespread. Finally, MglA and MglB homologues are also widespread in prokaryots [39], many of which are not motile, suggesting that small GTPase switches also regulate multiple biological processes in bacteria, a field of research that has not received the attention it deserves.

## Materials and Methods

### Bacterial Strains, Plasmids, and Growth

See Table S1 for plasmids and Table S2 for strains and their mode of construction. Primer sequences and plasmid construction schemes are provided in Tables S3 and S4. *M. xanthus* strains were grown at 32°C in CYE rich media as previously described [6]. Plasmids were introduced in *M. xanthus* by electroporation. Mutants and transformants were obtained by homologous recombination based on a previously reported method [6]. Complementation, expression of the fusion and mutant protein were all obtained after ectopic integration of the genes of interest at the Mx8-phage attachment site in appropriate deletion backgrounds (Table S2). For co-expression of both MglA_Q82L_ and MglA_Q82L_-YFP, expression of MglA_Q82L_ was additionally driven from the *car* locus, another ectopic site with the pCT2 system (Table 1 in [40]). Both integration at Mx8_att_ and *car* have no effect on cell motility [14],[40].

For phenotypic assays, cells, at a concentration of 4×10^9^ cfu ml^−1^, were spotted on CF-agar plates or CYE plates containing an agar concentration of 1.5%, incubated at 32°C, and photographed after 48 h with an Olympus SZ61 binocular or a Nikon Eclipse (model TE2000E) microscope.

### Expression and Purification of MglA and MglB

MglA-His_6_ and His_6_-MglB were expressed from the expression vector pET28(a) (Novagen). Expression of the recombinant proteins was induced in both cases by growing cells at room temperature for 20 h in the presence of 0.5 mM IPTG (isopropyl-h-d-thiogalactopyranoside). Cells were then harvested by centrifugation at 8,000 rpm for 10 min, resuspended in a buffer containing 50 mM NaH_2_PO_4_ pH 8.0, 300 mM NaCl, 10 mM imidazol, and lysed with a French press. The lysates were centrifuged twice (18,000 rpm, 4°C, 30 min) to remove debris prior to the purification. Supernatants were incubated with Nickel beads for 1 h at 4°C and the beads were collected and loaded into 5 ml HisTrap™ nickel columns (GE Healthcare). The elution was performed by using a buffer containing 50 mM NaH_2_PO_4_ pH 8.0, 300 mM NaCl, 250 mM imidazol, and for MglA, GDP 30 µM. Eluates were finally dialysed against a storage solution containing 50 mM NaH_2_PO_4_ pH 8.0, 300 mM NaCl, 10% Glycerol, and for MglA, GDP 30 µM. Protein purity and stability was determined by a Bradford assay and SDS-PAGE. The recombinant proteins were used both to immunize rabbits and perform biochemical in vitro assays.

### GTPγS-Binding Assay

Purified MglA, MglA_Q82L_, and MglB (1 µM final) were incubated at 30°C with either 20 µM non-hydrolysable [^35^S]GTPγS or 15 µM γ[^32^P]GTP in 50 mM HEPES (pH 7.5), 100 mM KCl, 1 mM MgCl_2_, 5 mM Pi, and 1 mM DTT. We found that, in solution, MglA is stabilized by addition of lipids; thus, all biochemical assays were conducted in the presence of 1 g/l Azolectin vesicles. Samples of 25 µl were filtered at the indicated times and radioactivity was counted [41]. The curves were obtained by fitting the data to the model y = A_o_(1−e^−kt^) with k = 0.29 min^−1^ for MglA alone and k = 0.12 min^−1^ for MglA in the presence of MglB.

### GTPase Assay

Purified MglA and MglA_Q82L_ (1 µM final) were loaded with 15 mM [γ-^32^P]GTP (∼1,400 cpm/pmoles) in 50 mM HEPES (pH 7.5), 100 mM KCl, 1 mM MgCl_2_, 5 mM Pi, 1 mM DTT, in the presence of 1 g/l Azolectin (Sigma) vesicles for 4 min at 30°C. The GTP hydrolysis was initiated by the addition of 1 µM (unless otherwise stated) of purified MglB. At the indicated times, aliquots of 25 µl were removed. ^32^Pi release was measured by the charcoal method [42]. Briefly, the 25 µl samples were added to 750 µl of 5%(w/v) charcoal (100–400 mesh, Sigma) in 50 mM NaH_2_PO_4_ (4°C) and vortexed. The charcoal was removed by centrifugation (5 min at 13.2 krpm) and the amount of radioactivity present in the supernatant was determined by liquid scintillation counting.

### Western Blotting

Western blotting was performed as previously described [40] with 1/10,000 dilutions of MglA or MglB antisera.

### Time Lapse Fluorescence Microscopy

Time lapse experiments were performed as previously described [43]. Microscopic analysis was performed using an automated and inverted epifluorescence microscope TE2000-E-PFS (Nikon, France). The microscope is equipped with “The Perfect Focus System” (PFS) that automatically maintains focus so that the point of interest within a specimen is always kept in sharp focus at all times, in spite of any mechanical or thermal perturbations. Images were recorded with a CoolSNAP HQ 2 (Roper Scientific, Roper Scientific SARL, France) and a 40×/0.75 DLL “Plan-Apochromat” or a 100×/1.4 DLL objective. All fluorescence images were acquired with a minimal exposure time to minimize bleaching and phototoxicity effects.

Cell tracking was performed automatically using a previously described macro under the METAMORPH software (Molecular devices); when appropriate, manual measurements were also performed to correct tracking errors with tools built into the software. Images were processed under both ImageJ 1.40g (National Institute of Health, USA) and METAMORPH.

### Statistical Analysis

Cells (*n*>100) were automatically segmented by successive morphological operations: h-dome extraction, gray-scale reconstruction, binary images, and morphological opening. To optimize segmentation, binary frames were sometimes corrected manually with appropriate tools built into the software. A binary mask was then used to perform integrated morphometric analysis and cell tracking. Cell tracking was performed following standard mathematical procedures already described in [43]. Computational scoring of cell reversals was obtained by tracking cells that showed clear directional changes, moving at least a 10^th^ of their cellular length in the opposite direction. To correlate these reversals to changes in fluorescence at the cell poles, the cell poles were automatically detected using a custom automation script (Visual Basic) under Metamorph 7.5 (Molecular Devices, Molecular Devices France, France). In this system, polar fluorescence inversions were systematically scored when the difference between the average grey intensity values of the poles became significantly different from the standard deviation of the average intensity value along the length of the cell. All selected cells were verified manually to ensure that the automatic process always scored actual reversals and polar fluorescence inversions. The cross-correlation coefficient (Rxy) between scored reversals and fluorescence pole-to-pole switchings for a time of delay (m) was calculated with the following equation:
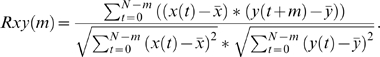
The maximum value is Rxy = 1 for a perfect correlation. The time lapse movies are composed of 30 s time frames due to illumination constraints (toxicity and bleaching). In these conditions, a Rxy>0.5 for a time delay = 0 (±30 s) means that the two events, fluorescence polar inversions (x(t)) and cellular reversals (y(t)), are highly correlated.

## Supporting Information

Figure S1 **Complementation of the Δ** ***mglB*** **and Δ** ***mglA*** **deletions.** The *mglB* deletion was complemented by integration of *mglB* or *mglB-yfp* at the Mx8 phage attachment site (see Methods). Hard agar motility assays show complete restoration of motility in both cases. Scale bar = 1 mm. (B) Complementation of the *mglA* deletion. Expression of *mglA* from Mx8 phage attachment site fully restores motility of an *mglA* deletion mutant.
(8.28 MB PDF)

Figure S2 ***mglB*** **is epistatic over** ***frzE*** **.** Agar motility phenotypes of Δ*frzE*, Δ*mglB*, and Δ*frzE* Δ*mglB* mutant strain. The Δ*frzE* Δ*mglB* and the Δ*mglB* mutants look identical in these assays. Note the different scales. Scale bar = 1 mm for the 1.5 Agar micrographs and 2 cm for the soft agar micrographs.
(2.37 MB PDF)

Figure S3 **Motility and developmental phenotypes of the MglA-YFP^m^ expressing strain.** Expression of MglA-YFP alone leads to motility defects that are especially observable on soft agar and during development on the TPM starvation medium. On the contrary, a strain expressing both MglA and MglA-YFP is indistinguishable from the WT strain in all assays, including the formation of fruiting bodies. Note the different scales. Scale bar = 1 mm for the 1.5 Agar and TPM micrographs and 2 cm for the soft agar micrographs.
(6.16 MB PDF)

Figure S4 **Box plot representations of MglB (A) and MglA (B) localization as a function of direction.** Each dark line represents polar fluorescence relationships for a same cell.
(0.16 MB PDF)

Figure S5 MglA-YFP dynamics are regulated by the Frz pathway. Oscillations of MglA-YFP in a *frzCD^c^* mutant. Fluorescence and corresponding phase contrast overlays are shown. Arrows indicate the direction of movement. Scale bar = 2 µm.
(0.41 MB PDF)

Figure S6 **Construction and characterization of MglA_Q82L_.** (A) Multiple protein sequence alignment and position of the MglA_Q82L_ substitution. The amino acid sequences of MglA, Arf6 (*homo sapiens*), and Cdc42 (*Saccharomyces cerevisiae*) were aligned using the ClustalW algorithm. The position of the Q82L substitution is marked in red. (B) MglA_Q82L_ binds but does not hydrolyze GTP. Time course of γ[^32^P]GTP binding to 1 µM of MglA or MglA_Q82L_ in the presence of MglB (1 µM) as described in the experimental procedures.
(0.24 MB PDF)

Figure S7 **MglA_Q82L_ is stably expressed as judged by anti-MglA Western blotting.**
(0.09 MB PDF)

Figure S8 **AglZ-YFP dynamics during cellular reversals in WT cells.** At the time of reversal, AglZYFP mostly localizes to the leading pole and switches to the new leading pole (white arrow). Note the absence of significant AglZ accumulation at the lagging cell pole. Time is shown in min. Scale bar = 2 µm.
(0.08 MB PDF)

Figure S9 **FrzS-YFP does not oscillate from pole to pole in absence of MglB.** (A) FrzS-YFP oscillations in WT cells. The white arrow points to FrzS-YFP switching to the new leading cell pole upon cellular reversal. (B) FrzS-YFP oscillations in the *mglB* mutant. Note the complete absence of FrzS-YFP inversion at the time of reversal. Time is shown in min.
(0.16 MB PDF)

Figure S10 **RomR-GFP does not oscillate in absence of** ***mglB*** **.** Time is shown in min.
(0.09 MB PDF)

Protocol S1 **Construction of MglA-YFP and MglA_Q82L_-YFP expressing strains.**
(0.05 MB DOC)

Table S1 **Plasmids.**
(0.07 MB DOC)

Table S2 ***Myxococcus*** **strains.**
(0.06 MB DOC)

Table S3 **Primers.**
(0.05 MB DOC)

Table S4 **Plasmid constructions.**
(0.04 MB DOC)

## Abbreviations

GAPs: GTPase Activating Proteins
GEFs: Guanine nucleotide Exchange Factors

## References

[1] Janetopoulos C, Firtel R. A (2008). Directional sensing during chemotaxis.. FEBS Lett.

[2] Charest P. G, Firtel R. A (2007). Big roles for small GTPases in the control of directed cell movement.. Biochem J.

[3] Wadhams G. H, Armitage J. P (2004). Making sense of it all: bacterial chemotaxis.. Nat Rev Mol Cell Biol.

[4] Mignot T, Kirby J. R (2008). Genetic circuitry controlling motility behaviors of Myxococcus xanthus.. Bioessays.

[5] Berleman J. E, Scott J, Chumley T, Kirby J. R (2008). Predataxis behavior in Myxococcus xanthus.. Proc Natl Acad Sci U S A.

[6] Bustamante V. H, Martinez-Flores I, Vlamakis H. C, Zusman D. R (2004). Analysis of the Frz signal transduction system of Myxococcus xanthus shows the importance of the conserved C-terminal region of the cytoplasmic chemoreceptor FrzCD in sensing signals.. Mol Microbiol.

[7] Sun H, Zusman D. R, Shi W. Y (2000). Type IV pilus of Myxococcus xanthus is a motility apparatus controlled by the frz chemosensory system.. Curr Biol.

[8] Li Y, Sun H, Ma X, Lu A, Lux R (2003). Extracellular polysaccharides mediate pilus retraction during social motility of Myxococcus xanthus.. Proc Natl Acad Sci U S A.

[9] Mignot T, Shaevitz J. W (2008). Active and passive mechanisms of intracellular transport and localization in bacteria.. Curr Opin Microbiol.

[10] Hodgkin J, Kaiser D (1979). Genetics of gliding motility in myxococcus-xanthus (myxobacterales) - 2 gene systems control movement.. Molecular & General Genetics.

[11] Bulyha I, Schmidt C, Lenz P, Jakovljevic V, Hone A (2009). Regulation of the type IV pili molecular machine by dynamic localization of two motor proteins.. Mol Microbiol.

[12] Mignot T, Merlie J. P, Zusman D. R (2005). Regulated pole-to-pole oscillations of a bacterial gliding motility protein.. Science.

[13] Mignot T, Shaevitz J. W, Hartzell P. L, Zusman D. R (2007). Evidence that focal adhesion complexes power bacterial gliding motility.. Science.

[14] Leonardy S, Freymark G, Hebener S, Ellehauge E, Sogaard-Andersen L (2007). Coupling of protein localization and cell movements by a dynamically localized response regulator in Myxococcus xanthus.. Embo J.

[15] Inclan Y. F, Vlamakis H. C, Zusman D. R (2007). FrzZ, a dual CheY-like response regulator, functions as an output for the Frz chemosensory pathway of Myxococcus xanthus.. Mol Microbiol.

[16] Inclan Y. F, Laurent S, Zusman D. R (2008). The receiver domain of FrzE, a CheA-CheY fusion protein, regulates the CheA histidine kinase activity and downstream signalling to the A- and S-motility systems of Myxococcus xanthus.. Mol Microbiol.

[17] Igoshin O. A, Goldbeter A, Kaiser D, Oster G (2004). A biochemical oscillator explains several aspects of Myxococcus xanthus behavior during development.. Proc Natnl Acad Sci U S A.

[18] Mauriello E. M, Mouhamar F, Nan B, Ducret A, Dai D (2010). Bacterial motility complexes require the actin-like protein, MreB and the Ras homologue, MglA.. Embo J.

[19] Yang R. F, Bartle S, Otto R, Stassinopoulos A, Rogers M (2004). AglZ is a filament-forming coiled-coil protein required for adventurous gliding motility of Myxococcus xanthus.. Journal of Bacteriology.

[20] Bernards A, Settleman J (2004). GAP control: regulating the regulators of small GTPases.. Trends Cell Biol.

[21] Bos J. L, Rehmann H, Wittinghofer A (2007). GEFs and GAPs: critical elements in the control of small G proteins.. Cell.

[22] Hartzell P, Kaiser D (1991). Upstream gene of the mgl operon controls the level of MglA protein in Myxococcus xanthus.. J Bacteriol.

[23] Koonin E. V, Aravind L (2000). Dynein light chains of the Roadblock/LC7 group belong to an ancient protein superfamily implicated in NTPase regulation.. Curr Biol.

[24] Hartzell P, Kaiser D (1991). Function of MglA, a 22-kilodalton protein essential for gliding in Myxococcus xanthus.. J Bacteriol.

[25] Blackhart B. D, Zusman D. R (1985). “Frizzy” genes of Myxococcus xanthus are involved in control of frequency of reversal of gliding motility.. Proc Natl Acad Sci U S A.

[26] Klein S, Franco M, Chardin P, Luton F (2006). Role of the Arf6 GDP/GTP cycle and Arf6 GTPase-activating proteins in actin remodeling and intracellular transport.. J Biol Chem.

[27] Anderson D. C, Gill J. S, Cinalli R. M, Nance J (2008). Polarization of the C. elegans embryo by RhoGAP-mediated exclusion of PAR-6 from cell contacts.. Science.

[28] Tong Z, Gao X. D, Howell A. S, Bose I, Lew D. J (2007). Adjacent positioning of cellular structures enabled by a Cdc42 GTPase-activating protein-mediated zone of inhibition.. J Cell Biol.

[29] Zhang S, Charest P. G, Firtel R. A (2008). Spatiotemporal regulation of Ras activity provides directional sensing.. Curr Biol.

[30] Lam H, Schofield W. B, Jacobs-Wagner C (2006). A landmark protein essential for establishing and perpetuating the polarity of a bacterial cell.. Cell.

[31] Huitema E, Pritchard S, Matteson D, Radhakrishnan S. K, Viollier P. H (2006). Bacterial birth scar proteins mark future flagellum assembly site.. Cell.

[32] Antonny B, Bigay J, Casella J. F, Drin G, Mesmin B (2005). Membrane curvature and the control of GTP hydrolysis in Arf1 during COPI vesicle formation.. Biochem Soc Trans.

[33] Ramamurthi K. S, Lecuyer S, Stone H. A, Losick R (2009). Geometric cue for protein localization in a bacterium.. Science.

[34] Ramamurthi K. S, Losick R (2009). Negative membrane curvature as a cue for subcellular localization of a bacterial protein.. Proc Natl Acad Sci U S A.

[35] Finlay B. B (2005). Bacterial virulence strategies that utilize Rho GTPases.. Curr Top Microbiol Immunol.

[36] Bowman A. B, Patel-King R. S, Benashski S. E, McCaffery J. M, Goldstein L. S (1999). Drosophila roadblock and Chlamydomonas LC7: a conserved family of dynein-associated proteins involved in axonal transport, flagellar motility, and mitosis.. J Cell Biol.

[37] Kappe S, Bruderer T, Gantt S, Fujioka H, Nussenzweig V (1999). Conservation of a gliding motility and cell invasion machinery in Apicomplexan parasites.. J Cell Biol.

[38] Sibley L. D (2004). Intracellular parasite invasion strategies.. Science.

[39] Dong J. H, Wen J. F, Tian H. F (2007). Homologs of eukaryotic Ras superfamily proteins in prokaryotes and their novel phylogenetic correlation with their eukaryotic analogs.. Gene.

[40] Mignot T, Merlie J. P, Zusman D. R (2007). Two localization motifs mediate polar residence of FrzS during cell movement and reversals of Myxococcus xanthus.. Mol Microbiol.

[41] Franco M, Chardin P, Chabre M, Paris S (1996). Myristoylation-facilitated binding of the G protein ARF1GDP to membrane phospholipids is required for its activation by a soluble nucleotide exchange factor.. J Biol Chem.

[42] Ferguson K. M, Higashijima T, Smigel M. D, Gilman A. G (1986). The influence of bound GDP on the kinetics of guanine nucleotide binding to G proteins.. J Biol Chem.

[43] Ducret A, Maisonneuve E, Notareschi P, Grossi A, Mignot T (2009). A microscope automated fluidic system to study bacterial processes in real time.. PLoS One.

