# Correction: A Bacterial Ras-Like Small GTP-Binding Protein and Its Cognate GAP Establish a Dynamic Spatial Polarity Axis to Control Directed Motility

**DOI:** 10.1371/annotation/ecce3f2a-35e6-4c27-b444-528248982dbf

**Published:** 2010-09-21

**Authors:** Yong Zhang, Michel Franco, Adrien Ducret, Tâm Mignot

The following information was missing from the Funding section: YZ is supported by a China Scholarship Council fellowship (#2008622014).

