# Supplementary figures and images for: A Bacterial Ras-Like Small GTP-Binding Protein and Its Cognate GAP Establish a Dynamic Spatial Polarity Axis to Control Directed Motility
Source: PLoS Biol. 2010 Jul 20;8(7):e1000430. doi: 10.1371/journal.pbio.1000430 (PMC2907295; doi:10.1371/journal.pbio.1000430)

**A****WT** **$\Delta mglB$**  **$mglB_{mx8}$**  **$mglB\text{-}YFP_{mx8}$** **1.5% Agar**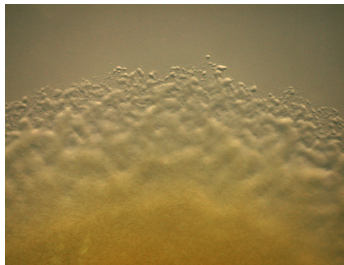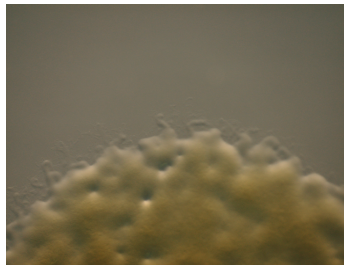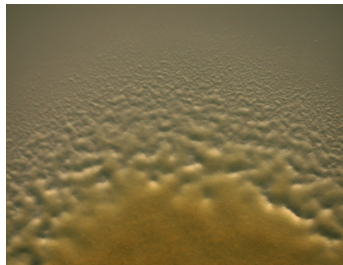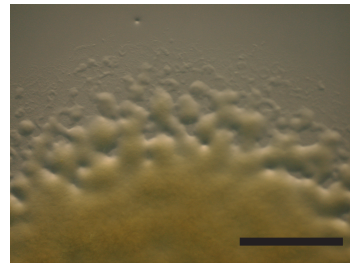**B****WT** **$\Delta mglA$**  **$mglA_{mx8}$** **1.5% Agar**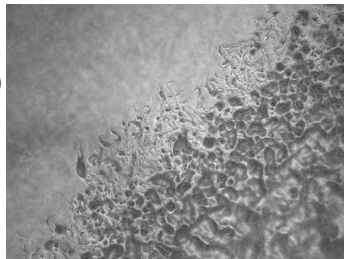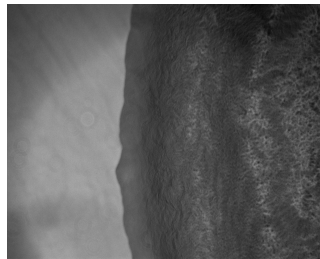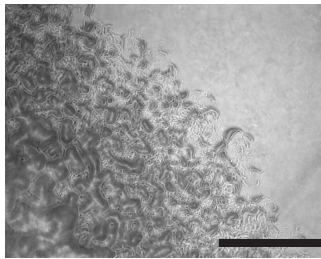

Supplement: Figure S1 — Complementation of the Δ mglB and Δ mglA deletions. The mglB deletion was complemented by integration of mglB or mglB-yfp at the Mx8 phage attachment site (see Methods). Hard agar motility assays show complete restoration of motility in both cases. Scale bar = 1 mm. (B) Complementation of the mglA deletion. Expression of mglA from Mx8 phage attachment site fully restores motility of an mglA deletion mutant. (8.28 MB PDF) [file pbio.1000430.s001.pdf]

**$\Delta frzE$**

**$\Delta mglB$**

**$\Delta frzE \Delta mglB$**

**1.5% Agar**

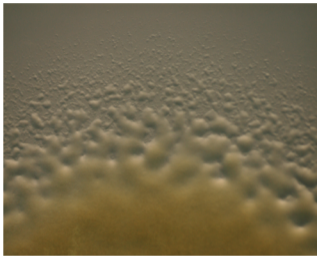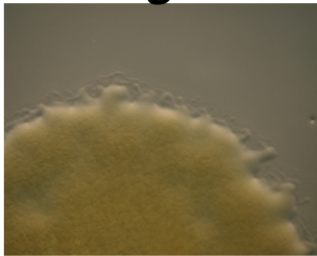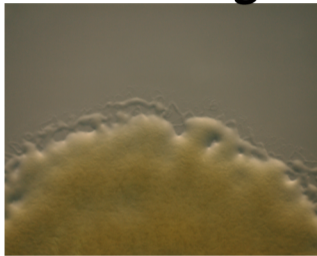

**0.5% Agar**

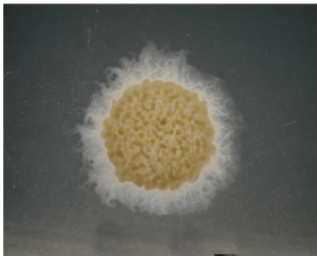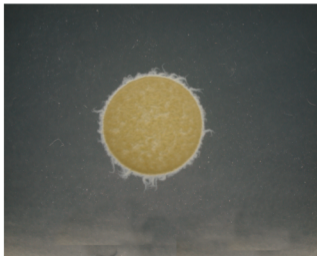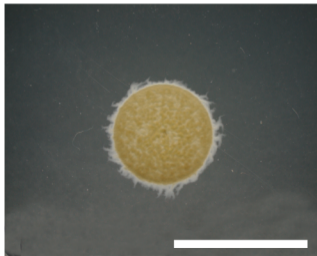

Supplement: Figure S2 — mglB is epistatic over frzE . Agar motility phenotypes of ΔfrzE, ΔmglB, and ΔfrzE ΔmglB mutant strain. The ΔfrzE ΔmglB and the ΔmglB mutants look identical in these assays. Note the different scales. Scale bar = 1 mm for the 1.5 Agar micrographs and 2 cm for the soft agar micrographs. (2.37 MB PDF) [file pbio.1000430.s002.pdf]

**MgIA**

**MgIA-YFP**

**MgIAYFP**

**WT**

**0.5% Agar**

**1.5% Agar**

**TPM**

DZ2

DZ2 *mgIAYFP*

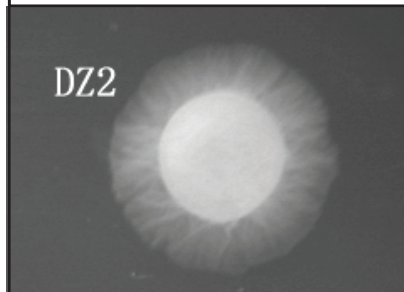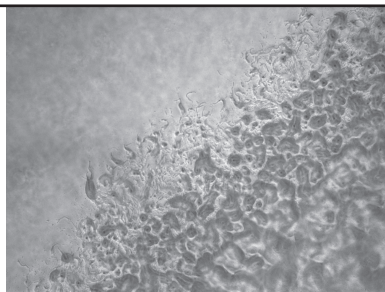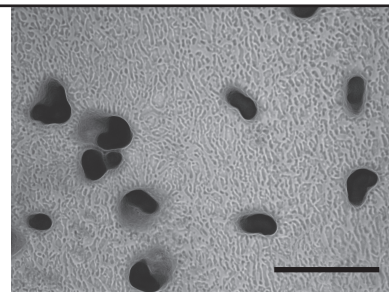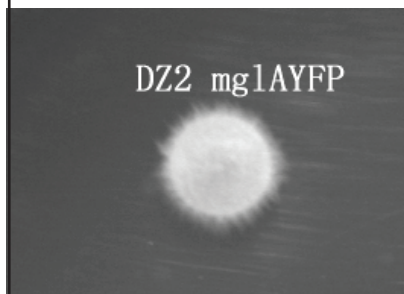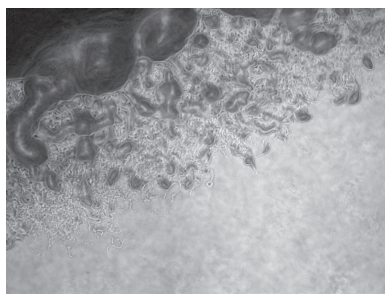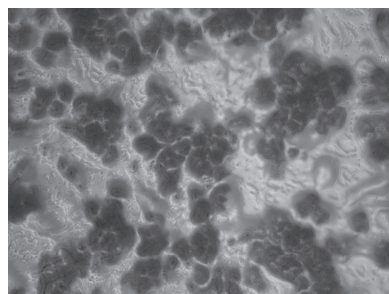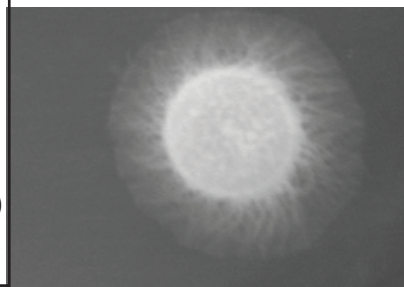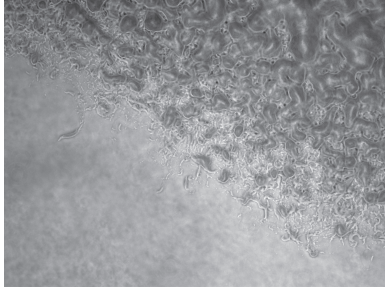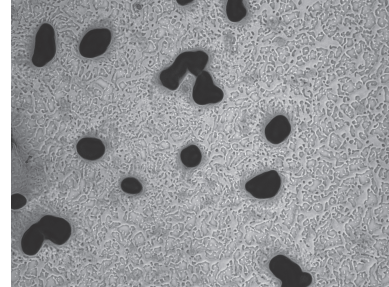

Supplement: Figure S3 — Motility and developmental phenotypes of the MglA-YFPm expressing strain. Expression of MglA-YFP alone leads to motility defects that are especially observable on soft agar and during development on the TPM starvation medium. On the contrary, a strain expressing both MglA and MglA-YFP is indistinguishable from the WT strain in all assays, including the formation of fruiting bodies. Note the different scales. Scale bar = 1 mm for the 1.5 Agar and TPM micrographs and 2 cm for the soft agar micrographs. (6.16 MB PDF) [file pbio.1000430.s003.pdf]

**A**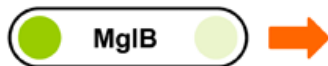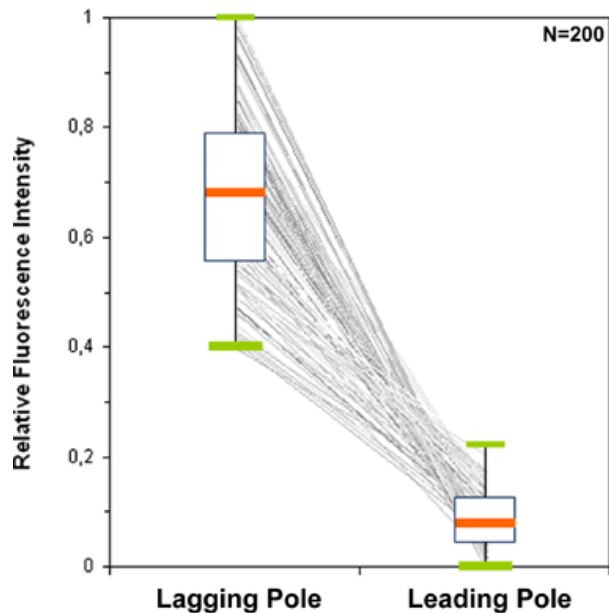**B**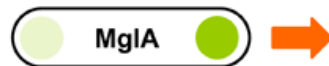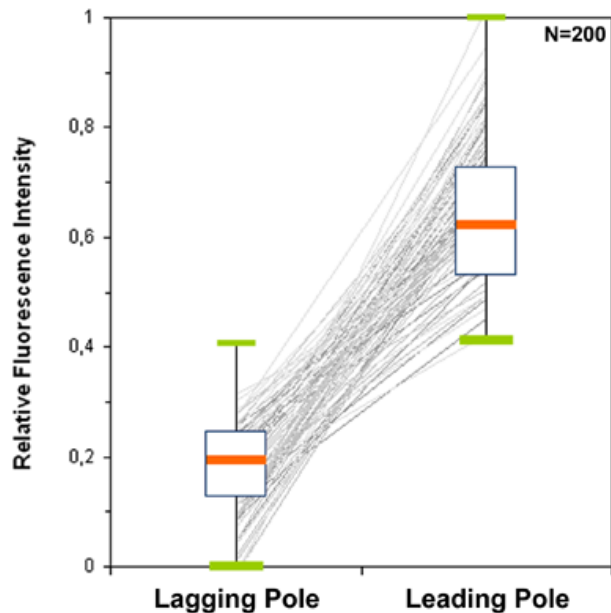

Supplement: Figure S4 — Box plot representations of MglB (A) and MglA (B) localization as a function of direction. Each dark line represents polar fluorescence relationships for a same cell. (0.16 MB PDF) [file pbio.1000430.s004.pdf]

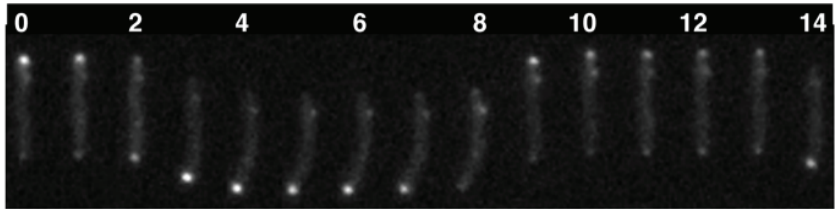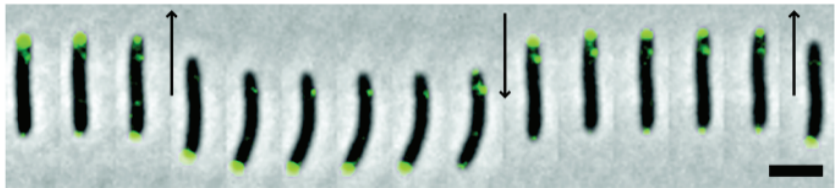

Supplement: Figure S5 — MglA-YFP dynamics are regulated by the Frz pathway. Oscillations of MglA-YFP in a frzCDc mutant. Fluorescence and corresponding phase contrast overlays are shown. Arrows indicate the direction of movement. Scale bar = 2 µm. (0.41 MB PDF) [file pbio.1000430.s005.pdf]

**WT**

***mglA*<sub>Q82L</sub>**

**αMglA**

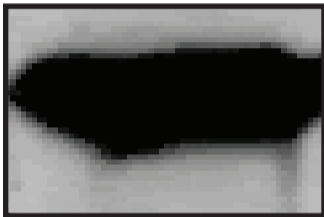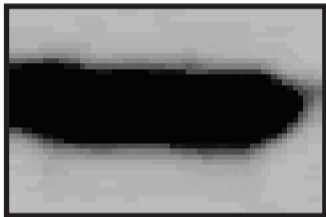

**Load**

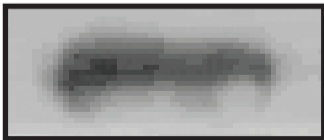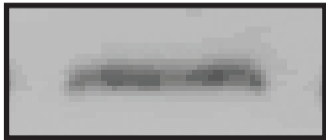

Supplement: Figure S7 — MglAQ82L is stably expressed as judged by anti-MglA Western blotting. (0.09 MB PDF) [file pbio.1000430.s007.pdf]

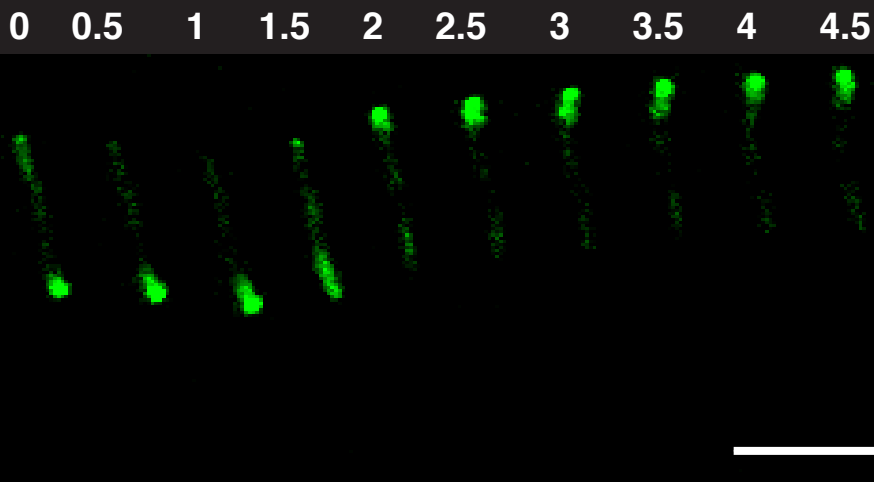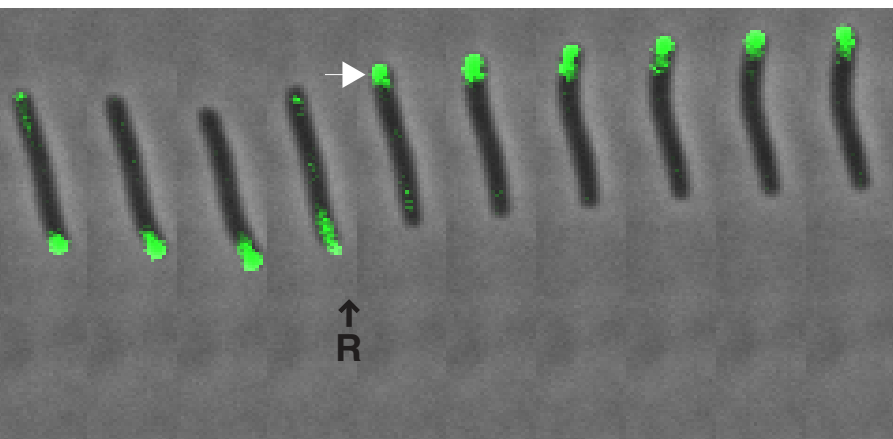

Supplement: Figure S8 — AglZ-YFP dynamics during cellular reversals in WT cells. At the time of reversal, AglZYFP mostly localizes to the leading pole and switches to the new leading pole (white arrow). Note the absence of significant AglZ accumulation at the lagging cell pole. Time is shown in min. Scale bar = 2 µm. (0.08 MB PDF) [file pbio.1000430.s008.pdf]

**A**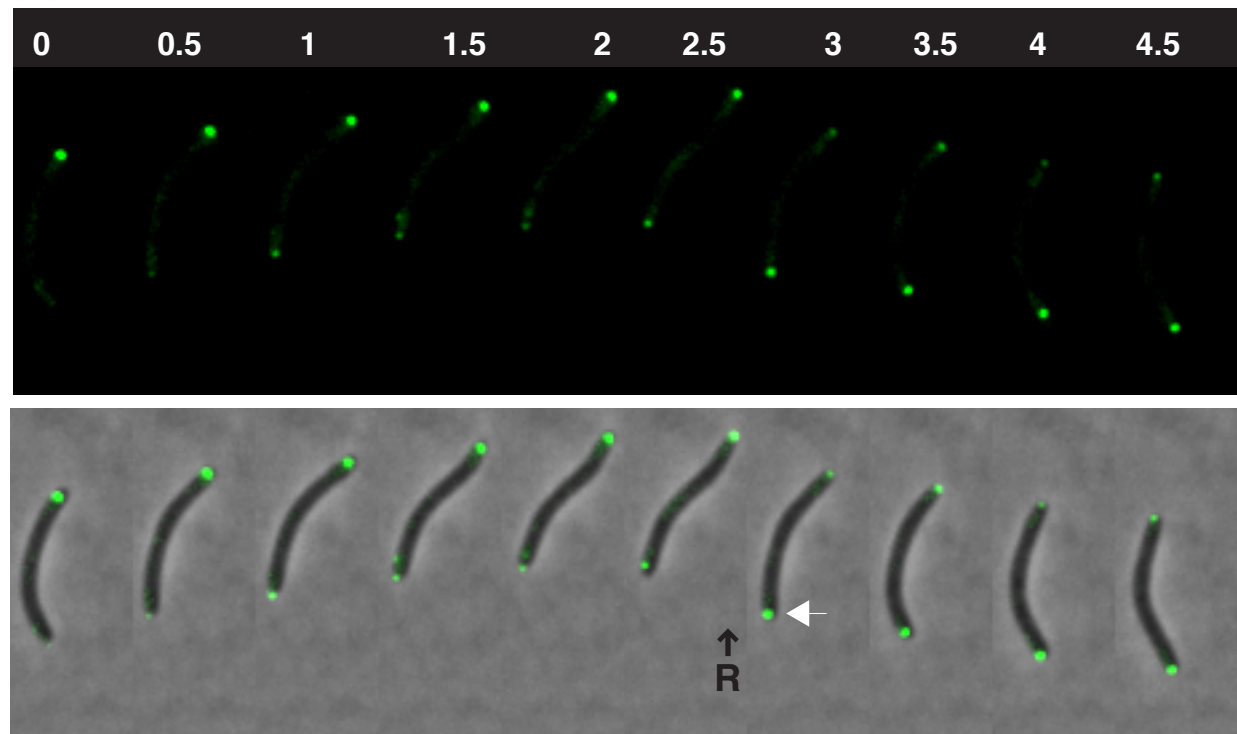**B**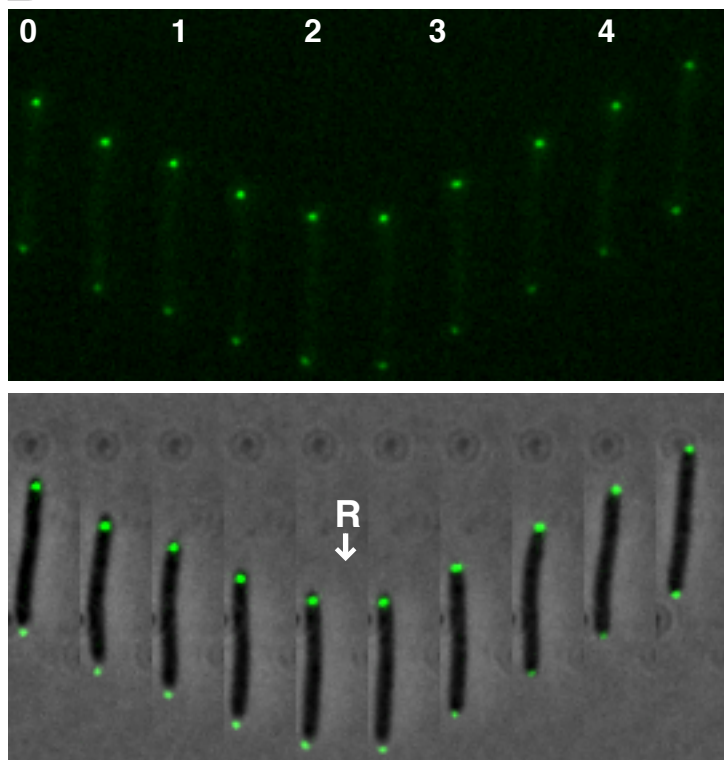

Supplement: Figure S9 — FrzS-YFP does not oscillate from pole to pole in absence of MglB. (A) FrzS-YFP oscillations in WT cells. The white arrow points to FrzS-YFP switching to the new leading cell pole upon cellular reversal. (B) FrzS-YFP oscillations in the mglB mutant. Note the complete absence of FrzS-YFP inversion at the time of reversal. Time is shown in min. (0.16 MB PDF) [file pbio.1000430.s009.pdf]

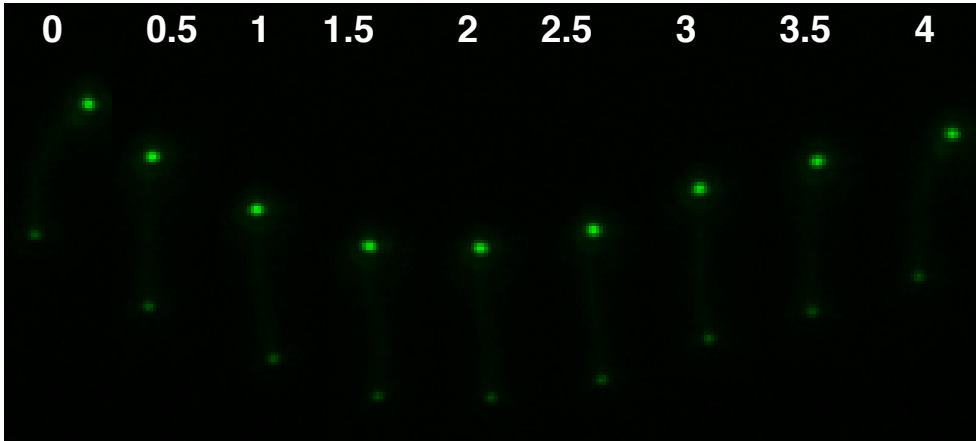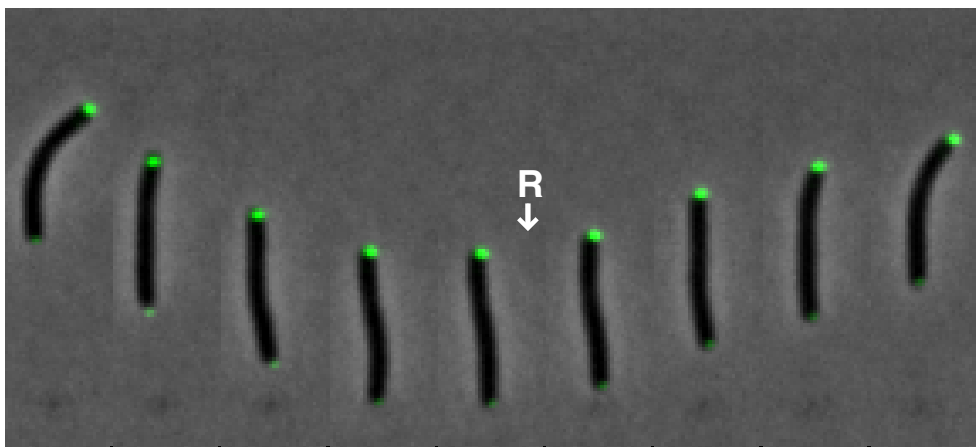

Supplement: Figure S10 — RomR-GFP does not oscillate in absence of mglB . Time is shown in min. (0.09 MB PDF) [file pbio.1000430.s010.pdf]
