# Supplementary material for: A Bacterial Ras-Like Small GTP-Binding Protein and Its Cognate GAP Establish a Dynamic Spatial Polarity Axis to Control Directed Motility
Source: PLoS Biol. 2010 Jul 20;8(7):e1000430. doi: 10.1371/journal.pbio.1000430 (PMC2907295; doi:10.1371/journal.pbio.1000430)
Supplement: Figure S6 — Construction and characterization of MglAQ82L. (A) Multiple protein sequence alignment and position of the MglAQ82L substitution. The amino acid sequences of MglA, Arf6 (homo sapiens), and Cdc42 (Saccharomyces cerevisiae) were aligned using the ClustalW algorithm. The position of the Q82L substitution is marked in red. (B) MglAQ82L binds but does not hydrolyze GTP. Time course of γ[32P]GTP binding to 1 µM of MglA or MglAQ82L in the presence of MglB (1 µM) as described in the experimental procedures. (0.24 MB PDF) [file pbio.1000430.s006.pdf]

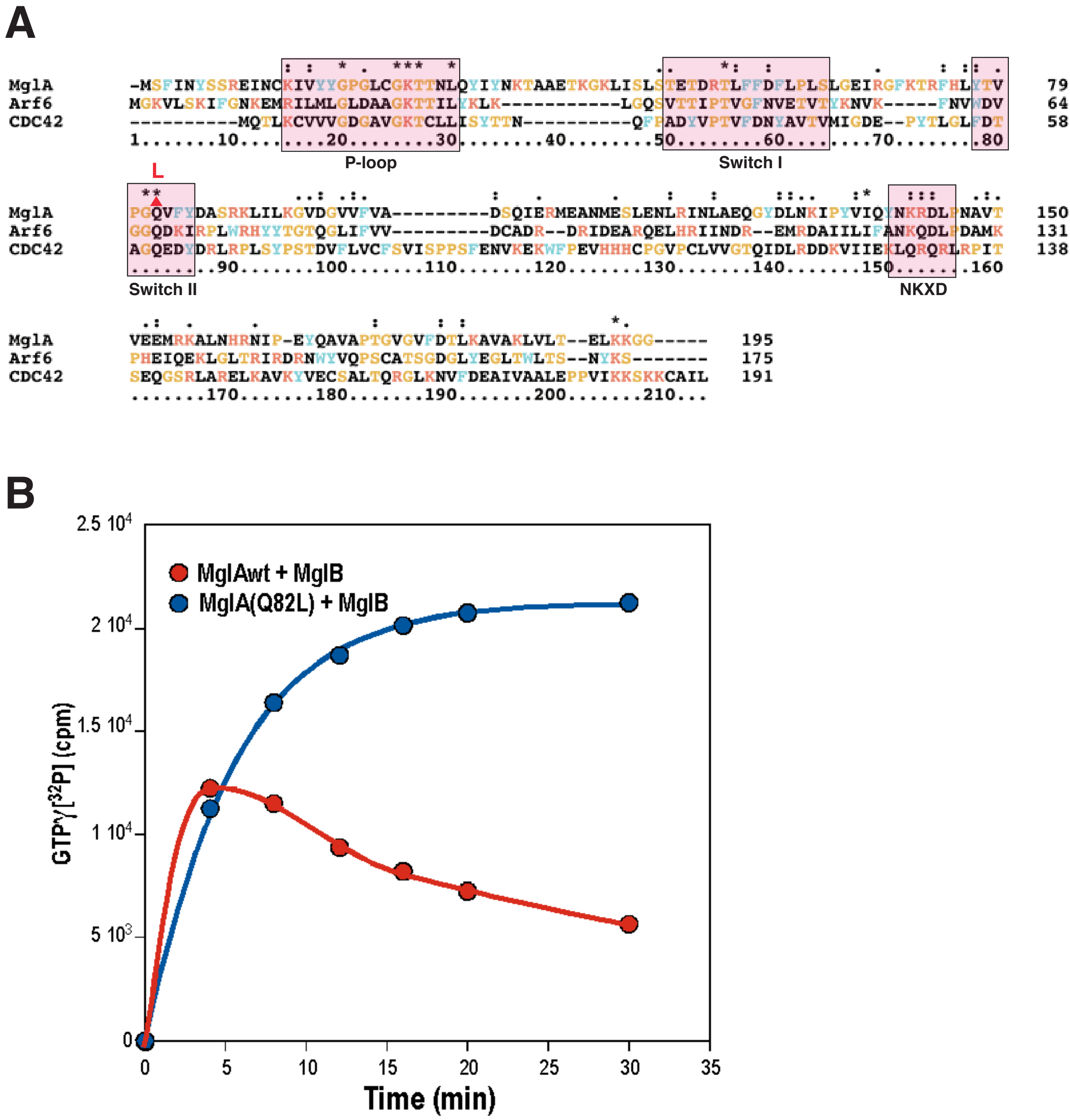
A
MglA -MSFINYSSREINCKIVYYGPGLCGKTTNLQYIYNKTAAETKGKLISLSTETDRTLFFDFLPLSLGEIRGFKTRFHLYTV 79
Arf6 MGKVLSKIFGNKEMRILMLGLDAAGKTTILYKLK-----------LGQSVTTIPTVGFNVETVTYKNVK-----FNVWDV 64
CDC42 ----------MQTLKCVVVGDGAVGKTCLLISYTTN----------QFPADYVPTVFDNYAVTVMIGDE--PYTLGLFDT 58
1.......10.......20.......30.......40.......50.......60.......70.......80
P-loop
Switch I
L
MglA PGQVFYDASRKLILKGVDGVVFVA---------DSQIERMEANMESLENLRINLAEQGYDLNKIPYVIQYNKRDLPNAVT 150
Arf6 GGQDKIRPLWRHYYTGTQGLIFVV---------DCADR--DRIDEARQELHRIINDR--EMRDAIILIFANKQDLPDAMK 131
CDC42 AGQEDYDRLRPLSYPSTDVFLVCFSVISPPSFENVKEKWFPEVHHHCPGVPCLVVGTQIDLRDDKVIIEKLQRQRLRPIT 138
.........90.......100.......110.......120.......130.......140.......150.......160
Switch II
NKXD
MglA VEEMRKALNHRNIP-EYQAVAPTGVGVFDTLKAVAKLVLT--ELKKGG----- 195
Arf6 PHEIQEKLGLTRIRDRNWYVQPSCATSGDGLYEGLTWLTS--NYKS------- 175
CDC42 SEQGSRLARELKAVKYVECSALTQRGLKNVFDEAIVAALEPPVIKKSKKCAIL 191
.......170.......180.......190.......200.......210...
B
MglAwt + MglB
MglA(Q82L) + MglB
$GTP^{\gamma}[^{32}P]$ (cpm)
$2.5\ 10^4$
$2\ 10^4$
$1.5\ 10^4$
$1\ 10^4$
$5\ 10^3$
0
0 5 10 15 20 25 30 35
Time (min)
