# Supplementary material for: A Bacterial Ras-Like Small GTP-Binding Protein and Its Cognate GAP Establish a Dynamic Spatial Polarity Axis to Control Directed Motility
Source: PLoS Biol. 2010 Jul 20;8(7):e1000430. doi: 10.1371/journal.pbio.1000430 (PMC2907295; doi:10.1371/journal.pbio.1000430)
Supplement: Protocol S1 — Construction of MglA-YFP and MglAQ82L-YFP expressing strains. (0.05 MB DOC) [file pbio.1000430.s011.doc]

**PROTOCOL S1**

**Construction of MglA-YFP^m^ and MglA_Q82L_-YFP^m^ expressing cells**

We first attempted to construct another MglA chimera where YFP is fused to the N-terminus of MglA, but this fusion proved completely dysfunctional (data not shown). Thus, we tested whether the co-expression of MglA and MglA-YFP could restore defects that are observed when MglA-YFP is expressed alone in place of MglA. When MglA and MglA-YFP were expressed together, swarming on hard and soft agar of the merodiploid cells was indistinguishable from the WT strain (Fig. S3). Also, the merodiploid cells reversed with a WT reversal frequency, showing that expression of MglA completely rescues the defect of the MglA-YFP fusion (data not shown). For clarity, merodiploid cells expressing MglA and MglA-YFP are referred to as MglA-YFP^m^ cells throughout the manuscript; the results suggest that MglA-YFP^m^ is an appropriate alternative to study the dynamics of MglA during the reversal cycle.

To analyze the dynamics of MglA_Q82L_-YFP, we could not use an MglA_Q82L_-YFP chimera expressed alone because the resulting construct was largely dysfunctional: the cells hardly showed detectable motility (data not shown). To overcome this problem, we constructed another merodiploid strain where MglA_Q82L_-YFP is expressed together with unmarked MglA_Q82L_: as expected, the resulting strain behaved like the strain expressing MglA_Q82L_ alone (data not shown).
