# Supplementary material for: A Bacterial Ras-Like Small GTP-Binding Protein and Its Cognate GAP Establish a Dynamic Spatial Polarity Axis to Control Directed Motility
Source: PLoS Biol. 2010 Jul 20;8(7):e1000430. doi: 10.1371/journal.pbio.1000430 (PMC2907295; doi:10.1371/journal.pbio.1000430)
Supplement: Table S1 — Plasmids. (0.07 MB DOC) [file pbio.1000430.s012.doc]

| Table S1. Plasmids |  |  |
| --- | --- | --- |
| Name | Description | Source |
| pBJ114  pSWU19  pSWU30  pCT2  pEYPN1  pEFrzSY  pBJAglZY  pBJDmglA  pBJmglAYR  pBJDMglB  pBJDmglBA  pSWU19mglB  pSWU19mglA  pSWU19mglBY  pSWU30mglAY  pSWU30mglA_Q82L_  pSWU19mglA_Q82L_  pCTmglA_Q82L_  pSWU30mglAQY  pBJMglBC | Used to create deletions, *galK*, Km^R^  Kan^R^ used to integrate genes ectopically at Mx8_att_  Tet^R^ used to integrate genes ectopically at Mx8_att_  Kan^R^ used to integrate genes ectopically at the *car* locus  Plasmid carrying the *yfp* gene  pEYFPN1 with a cassette allowing construction of the *frzS-yfp* chimeric gene  pBJ114 with a cassette allowing construction of the *aglZ-yfp* chimeric gene  pBJ114 with a deletion cassette for *mglA*  pBJ114 with a cassette allowing construction of the *mglA-yfp* chimeric gene  pBJ114 with a deletion cassette for *mglB*  pBJ114 with a deletion cassette for *mglBA*  pSWU19 allowing expression of *mglB* from its own promoter at Mx8_att_  pSWU19 allowing expression of *mglA* from its own promoter at Mx8_att_  pSWU19 allowing expression of *mglB-yfp* from its own promoter at Mx8_att_  pSWU30 allowing expression of *mglA-yfp* from its own promoter at Mx8_att_  pSWU30 allowing expression of *mglA_Q82L_* from its own promoter at Mx8_att_  pSWU30 allowing expression of *mglA_Q82L_* from its own promoter at Mx8_att_  pCT2 allowing expression of *mglA_Q82L_* from its own promoter at *car*  pSWU30 allowing expression of *mglA_Q82L_yfp* from its own promoter at Mx8_att_  pBJ114 allowing expression of *mglB-mCherry* from endogenous locus | [44]  L. Søgaard-Andersen  L. Søgaard-Andersen  [12]  Clontech  This work  [13]  [18]  [18]  This work  This work  This work  This work  This work  This work  This work  This work  This work  This work  This work |
