# Supplementary material for: A Bacterial Ras-Like Small GTP-Binding Protein and Its Cognate GAP Establish a Dynamic Spatial Polarity Axis to Control Directed Motility
Source: PLoS Biol. 2010 Jul 20;8(7):e1000430. doi: 10.1371/journal.pbio.1000430 (PMC2907295; doi:10.1371/journal.pbio.1000430)
Supplement: Table S2 — Myxococcus strains. (0.06 MB DOC) [file pbio.1000430.s013.doc]

| Table S2. *Myxococcus* strains | | | |
| --- | --- | --- | --- |
| Strain | Construction | Source | Genotype |
| DZ2  DZF3377  TM9  TM12  TM4  TM17  TM155  TM158  DZ4481  DZ4484  TM203  DZ4038  TM187  TM226  TM199  TM168  TM197  TM192  TM201  TM200  TM184  TM182  TM23  TM183  TM181  TM285  TM289  TM310  TM239  TM303  TM329  TM321  TM224  TM317  TM299  TM223  TM225  TM302 | Wild type  DZ2 *frzE:: tn5Ω226*  DZ2 *aglZ-yfp*  DZ2 *mglA*  DZ2 *frzS-gfp*  DZ2 *mglA-yfp*  DZ2 *∆mglB*  DZ2 *∆mglBA* (pBJDmglBA)  DZ2 *∆frzE*  DZ2 *∆frzZ*  DZ2 *romR-gfp*  DZ2 frzCD^c^ (frzCD::Tn5Ω224)  DZ4481 *∆mglB* (pBJDmglB)  DZ4484 *∆mglB* (pBJDmglB)  DZ4038 *∆mglB*  TM155 *mx8_att_::mglB* (pSWU19mglB)  TM12 *mx8_att_::mglA* (pSWU19mglA)  TM155 *mx8_att_::mglB-yfp* (pSWU19mglBY)  TM187 *mx8_att_::mglB-yfp* (pSWU19mglBY)  TM199 *mx8_att_::mglB-yfp* (pSWU19mglBY)  TM17 *mx8_att_::mglA* (pSWU19mglA)  DZ4481 *mx8_att_::mglA-yfp* (pSWU19mglAY)  DZ4038 *mglA-yfp* (pBJmglAYR)  TM23 *mx8_att_::mglA* (pSWU19mglA)  TM155 *mx8_att_::mglA-yfp* (pSWU30mglAY)  TM12 *mx8_att_::mglA_Q82L_* (pSWU30mglA_Q82L_)  TM158 *mx8_att_::mglA_Q82L_* (pSWU19mglA_Q82L_)  TM289 *frzE:: tn5Ω226*  TM12 *mx8_att_::mglA_Q82L_* (pSWU19mglA_Q82L_)  TM239 *frzE:: tn5Ω226*  TM12 *car::mglA_Q82L_* (pCTmglA_Q82L_)  TM329 *mx8_att_::mglA_Q82L_yfp* (pSWU30mglAQY)  TM155 *aglZ-yfp* (pBJAglZY)  TM285 *aglZ-yfp* (pBJAglZY)  TM285 *frzS-yfp* (pEFrzSY)  TM155 *frzS-yfp* (pEFrzSY)  TM155 *romR-gfp*  TM184 *mglB-mCherry* (pBJmglBC) | Laboratory collection  [45]  [13]  [18]  Mignot *et al*. (2005)  [18]  This work  This work  [6]  [6]  This work  [6]  This work  This work  This work  This work  This work  This work  This work  This work  This work  This work  This work  This work  This work  This work  This work  This work  This work  This work  This work  This work  This work  This work  This work  This work  This work  This work | WT  Δ*frzE*  *aglZ-yfp*  Δ*mglA*  *frzS-gfp*  *mglA-yfp*  Δ*mglB*  Δ*mglBA*  Δ*frzE*  Δ*frzZ*  *romR-gfp*  *frzCD^c^*  Δ*mglB* Δ*frzE*  Δ*mglB* Δ*frzZ*  Δ*mglB frzCD^c^*  Δ*mglB mglB*  Δ*mglA mglA*  Δ*mglB mglB-yfp*  *frzE* Δ*mglB mglB-yfp*  *frzCD^c^* Δ*mglB mglB-yfp*  *mglA-YFP mglA*  *frzE mglA-YFP mglA*  *frzCD^c^ mglA-YFP*  *frzCD^c^ mglA-YFP mglA*  Δ*mglB mglA-YFP mglA*  Δ*mglA mglA_Q82L_*  Δ*mglB* Δ*mglA mglA_Q82L_*  Δ*frzE* Δ*mglB* Δ*mglA mglA_Q82L_*  Δ*mglA mglA_Q82L_*  Δ*frzE* Δ*mglA mglA_Q82L_*  Δ*mglA mglA_Q82L_*  Δ*mglA mglA_Q82L_ mglA_Q82L_-yfp*  Δ*mglB aglZ-yfp*  Δ*mglA mglA_Q82L_ aglZ-yfp*  Δ*mglA mglA_Q82L_ frzS-yfp*  Δ*mglB frzS-yfp*  Δ*mglB romR-gfp*  *mglA-YFP mglA mglB-mCherry* |
