# Supplementary material for: A Bacterial Ras-Like Small GTP-Binding Protein and Its Cognate GAP Establish a Dynamic Spatial Polarity Axis to Control Directed Motility
Source: PLoS Biol. 2010 Jul 20;8(7):e1000430. doi: 10.1371/journal.pbio.1000430 (PMC2907295; doi:10.1371/journal.pbio.1000430)
Supplement: Table S3 — Primers. (0.05 MB DOC) [file pbio.1000430.s014.doc]

| Table S3. Primers | |  |
| --- | --- | --- |
| Plasmids | Name | Sequences of primers (5’---3’) |
| pBJDmglB | ∆MglB-1up  ∆MglB-1dwn  ∆MglB-2up  ∆MglB-2dwn | GGAATTCATCCTCCAGCGTCACCGTGT  TGGCTTCCCGGGTTACTCTTCGTACATCACC  TAACCCGGGAAGCCATGTCC  GGGGTACCCGTTCTTGTGGATGATGACG |
| pBJDmglBA | ∆MglBA-1up  ∆MglBA-1dwn  ∆MglBA-2up  ∆MglBA-2dwn | GGAATTCGTCCCATCCTCCAGCGTCAC  GGGCGCTTGCTTCAAGGGCGTATGCGCTCCT  TTGAAGCAAGCGCCCAGGCG  GGGGTACCTTCTCCTCGACGCCGAGCAG |
| pSWU19mglB | pSWUMGLBA fw  pSMglB R | GGAATTCGTGGGAAGGGCTCTTTCAGG  CCCAAGCTTTCCCGGGTTACTCGCTGAAG |
| pSWU19mglA | pSWUMGLBA fw  pSWUMGLBA rv | GGAATTCGTGGGAAGGGCTCTTTCAGG  CCCAAGCTTCGACACGCACGGTACGACCT |
| pSWU19mglBY pBJmglBC | pSWUMGLBA fw  pSMBCY 1R  pSMBCY 2F  pSMBCY 2R | GGAATTCGTGGGAAGGGCTCTTTCAGG  GCCCGGCGCGCCAGAGCTCGAGCCAGACTCGCTGAAGAGGTT  TCTGGCGCGCCGGGCATGGTGAGCAAGGGC  CCCAAGCTTTCCCGGGTTACTTGTACAGCTCGTC |
| pSWU30mglAY | pSMBACY F  pSMBACY R | CCATGATTACGAATTGTGGGAAGGGCTCTTTCAGG  GGCCAGTGCCAAGCTCGACACGCACGGTACGACCT |
| pSWU19mglA_Q82L_  pSWU30mglA_Q82L_ pSWU30mglAQY | pSWUMGLBA fw  MglA-Q82L-1R  MglA-Q82L-2F  pSWUMGLBA rv | GGAATTCGTGGGAAGGGCTCTTTCAGG  ACGAGACCGGGCACCGTGTACAG  ACACGGTGCCCGGTCTCGTCTTCTACGACGCCAG  CCCAAGCTTCGACACGCACGGTACGACCT |
| PCTmglA_Q82L_ | PCTAQ82L-F  PCTAQ82L-R | GGGGTACCGTGGGAAGGGCTCTTTCAGG  GGGGTACCCGACACGCACGGTACGACCT |
