# Supplementary material for: A Bacterial Ras-Like Small GTP-Binding Protein and Its Cognate GAP Establish a Dynamic Spatial Polarity Axis to Control Directed Motility
Source: PLoS Biol. 2010 Jul 20;8(7):e1000430. doi: 10.1371/journal.pbio.1000430 (PMC2907295; doi:10.1371/journal.pbio.1000430)
Supplement: Table S4 — Plasmid constructions. (0.04 MB DOC) [file pbio.1000430.s015.doc]

| Table S4. Plasmid constructions | |
| --- | --- |
| Plasmid | Construction scheme^a^ |
| pBJDmglB | Primer pairs ∆MglB-1up-∆MglB-1dwn and ∆MglB-2up-∆MglB-2dwn were used to amplify 1kb fragment upstream and downstream from the *mglB* open-reading frame and used to generate a 2kb fragment by SOE-PCR. This fragment was then ligated at the *Eco*RI and *Kpn*I sites of pBJ114. The resulted construct was sequenced to ensure the absence of PCR-introduced mutations. |
| pBJDmglBA | As described above ∆MglB-1up/ ∆MglB-1dwn and ∆MglBA-1up/ ∆MglBA-1dwn-amplified PCR products were used for SOE-PCR and cloning in PBJ114 at the *Eco*RI and *Kpn*I sites. |
| pSWU19mglB | A fragment encompassing *mglB* and the *mglBA* promoter region was amplified from the DZ2 chromosome with primers pSWUMGLBAfw-pSMglBR and cloned at the *Eco*RI and *Hind*III sites of pSWU19 |
| pSWU19mglA | A fragment encompassing *mglA* and the *mglBA* promoter region from the was amplified from the DZ2 *∆mglB* chromosome with primers pSWUMGLBAfw-pSWUMGLBArv and cloned at the *Eco*RI and *Hind*III sites of pSWU19. |
| pSWU19mglBY | *mglB* was amplified from the DZ2 chromosome with the pSWUMGLBAfw-pSMBCY1R primer pair and fused to a pSMBCY2F-pSMBCY2R *yfp* fragment amplified from the commercial plasmid pEYFP by SOE PCR. The resulting fragment was then ligated to the *EcoRI* and *HindIII* sites of pSWU19. |
| pSWU30mglAY | *mglA-yfp* was amplified from the TM159 chromosome with primer pair pSMBACYF-pSMBACYR. The resulting fragment was then cloned at the *EcoRI* and *HindIII* sites of pSWU30*.* |
| pSWU19mglA_Q82L_  pSWU30mglA_Q82L_ | *mglA_Q82L_* was generated by SOE PCR fusing a fragment amplified from primers pSWUMGLBA fw- MglA-Q82L-1R to a fragment amplified from MglA-Q82L-2F / pSWUMGLBA rv using plasmid pSWU19mglA as a template. The resulting fragment was then ligated to the *EcoRI* and *HindIII* sites of pSWU19/pSWU30. |
| pSWU30mglAQY | A fragment corresponding to mglAQ82L-yfp was obtained after fusing a fragment amplified with primers pSMBACY F-pSMBACY R from plasmid pSWU19mglA_Q82L_ and a fragment corresponding to the yfp sequence from pEYFP. The resulting fragment was then ligated to the *EcoRI* and *HindIII* sites of pSWU30. |
| pBJmglBC | Primers pSWUMGLBA fw/ pSMBCY 1R were used to amplify the fragment *mglB* and its promoter from the DZ2 chromosome. Primers pSMBCY 2F/ pSMBCY 2R were used to amplify the fragment *mCherry* from the commercial plasmid pmCherry-1. Both fragments were fused by SOE PCR and cloned at the *EcoRI* and *HindIII* sites of pBJ114 |
| PCTmglA_Q82L_ | A fragment corresponding to mglA_Q82L_ was amplified from pSWU19mglA_Q82L_ with primer pair PCTAQ82L-F/PCTAQ82L-R and cloned at the *Kpn*I site of pCT2 |

^a^ All plasmid inserts were sequenced to ensure the absence of PCR-introduced mutations.
